# Tau protein binds to the P53 E3 ubiquitin ligase MDM2

**DOI:** 10.1038/s41598-023-37046-8

**Published:** 2023-06-23

**Authors:** Martina Sola, Azucena Rendon-Angel, Viviana Rojo Martinez, Jacopo Sgrignani, Claudia Magrin, Ester Piovesana, Andrea Cavalli, Paolo Paganetti, Stéphanie Papin

**Affiliations:** 1 Laboratory for Aging Disorders, Laboratories for Translational Research, Ente Ospedaliero Cantonale, Room 102a, Via Chiesa 5, 6500 Bellinzona, Switzerland; 2 PhD Program in Neurosciences, Faculty of Biomedical Sciences, Università Della Svizzera Italiana, Lugano, Switzerland; 3 Computational Structural Biology, Institute for Research in Biomedicine, Università Della Svizzera Italiana, Bellinzona, Switzerland; 4 Swiss Institute of Bioinformatics, Lausanne, Switzerland; 5 Neurocentro Della Svizzera Italiana, Ente Ospedaliero Cantonale, Lugano, Switzerland

**Keywords:** Mechanisms of disease, Proteolysis

## Abstract

Tau gene mutations cause a progressive dementia and neurotoxic Tau forms deposited in neurofibrillary tangles are hallmarks of neurodegenerative tauopathies. Loss of non-canonical Tau functions may contribute to disease. In fact, Tau depletion affects the cellular response to DNA damage and tauopathies exhibit the accumulation of DNA lesions. Moreover, Tau modulates P53 activity and cell fate. Considering that MDM2 is the main antagonist of P53, we investigated, using orthogonal assays, if Tau interacts with MDM2. We report the existence in cells and brain of a Tau-MDM2 complex that, in vitro, exhibits reduced P53 ubiquitination activity in a manner sensitive to a Tau mutation. The Tau-MDM2 interaction involves the microtubule-binding domain of Tau and the acidic domain of MDM2, reminiscent of the binding of Tau to negatively charged microtubules. Notably, MDM2 accumulates aberrantly in neurofibrillary tangles. Aging-associated insults may expose a novel loss-of-function of Tau in neurodegeneration and cancer.

## Introduction

Self-assembly and deposition of the microtubule-associated protein Tau in brain is shared by a group of neurodegenerative disorders termed tauopathies^1^. Accumulation of pathogenic Tau forms in neurofibrillary tangles and neuropil threads correlates with the clinical course, although Tau deposits display disease-specific patterns of affected brain area and propagation^2^.

Alzheimer’s disease (AD) is the most common tauopathy, whereas rare disease forms include progressive supranuclear palsy, Pick’s disease, globular glial tauopathy, and others^3^. Notably, over forty autosomal dominant mutations in the *MAPT* gene encoding for Tau cause FTLD-Tau, a small group of progressive frontotemporal lobar or corticobasal degenerations^4,5^. Genetic variations impair predominantly Tau binding to microtubule and favor self-assembly. It is deduced that abnormal conformation and modification of Tau cause its dissociation from microtubules, its self-assembly and the initiation of a cascade of gain-of-toxic events leading to neuronal dysfunction, clinical symptoms and premature mortality^6^.

The microtubule-unrelated physiological roles of Tau render conceivable its loss-of-function contribution to disease. As an example, Tau localizes in the cell nucleus and heat or oxidative stress favor nuclear Tau translocation^7^. Tau binds to and protects the DNA^8^, so that Tau-KO neurons display enhanced DNA damage when compared to normal neurons^9^. Possibly related to this observation, chromosomal abnormalities and increased DNA damage are found in AD-derived fibroblasts^10–12^. We reported an increased nuclear translocation of Tau with a distinct phosphorylation pattern in cells exposed to a DNA damaging drug^13^.

This important function in DNA protection hint to an implication of Tau in cancer, in addition to neurodegeneration. Indeed, several studies described positive or negative correlations between Tau mRNA expression and survival in various types of cancers^14^ and *MAPT* mutations are associated to an increased risk of cancer^15^. Microtubule-related and non-canonical functions of Tau may underly these correlations. We described that in cells with an unresolved DNA damage, Tau modulates P53 stability and activity leading to a change in cell fate response^13^. Overall, these results suggest that the contribution of Tau loss-of-function to the pathogenesis of disease may require the concomitant exposure to (aging-associated) cellular insults.

Somatic mutations in the gene *TP53* encoding for the guardian of the genome P53 account for > 50% of human cancers^16,17^. Under basal conditions, cells maintain a low amount of P53 by post-translational mechanisms tightly regulated by a complex protein network mainly governed by the E3 ubiquitin-protein ligase MDM2^18^. All three structural domains of MDM2 participate in steering P53 degradation by the ubiquitin proteasome system^19^. The N-terminus of MDM2 forms a deep hydrophobic pocket binding to an α-helix in the transactivation domain of P53 thus antagonizing its target gene transcription^20^. The C-terminal RING domain of MDM2 executes the E3 ligase activity that ubiquitinates lysine residues at the C-terminus of P53^21,22^. The RING domain also contributes to tethering the MDM2-P53 complex to the 19S proteasome subunit^23^. When phosphorylated (mainly by CK1δ), the central acidic region of MDM2 participates to P53 binding^24^. However, when unphosphorylated, an intramolecular interaction between the central domain and the RING domain impairs the binding of this latter to the proteasome^24^. The P53-MDM2 axis is a fundamental pathway to overcome cellular stress due to its role as central coordinator of the cell response. As an example, P53-MDM2 drive the recovery process during the DNA damage response, but also balance survival against senescence or apoptosis of irreversibly lesioned cells^25^.

We now report the molecular mechanism inherent to Tau-dependent P53 modulation. The starting point of our study was the observation that Tau-depletion also modified MDM2 and that the effect of Tau on P53 was reversed by nutlin-3, a high affinity antagonistic ligand for the hydrophobic pocket of MDM2 binding to P53^13^. We show herein that Tau binds to MDM2 and that this inhibits P53 ubiquitination in vitro in a manner sensitive to the presence of the P301L mutation linked to FTLD-Tau. Notably, we also observed an abnormal accumulation of MDM2 in Tau tangles of the AD brain. The protein regions interacting with each other in the Tau-MDM2 complex correspond to the microtubule-binding domain (MBD) of Tau and the central acidic domain of MDM2, an interaction that is reminiscent to that of the positively charged MBD with the negatively charged microtubule surface.

## Results

### Tau binds to MDM2 in HeLa and SH-SY5Y cells, and in human brain tissue

**Figure 1:**
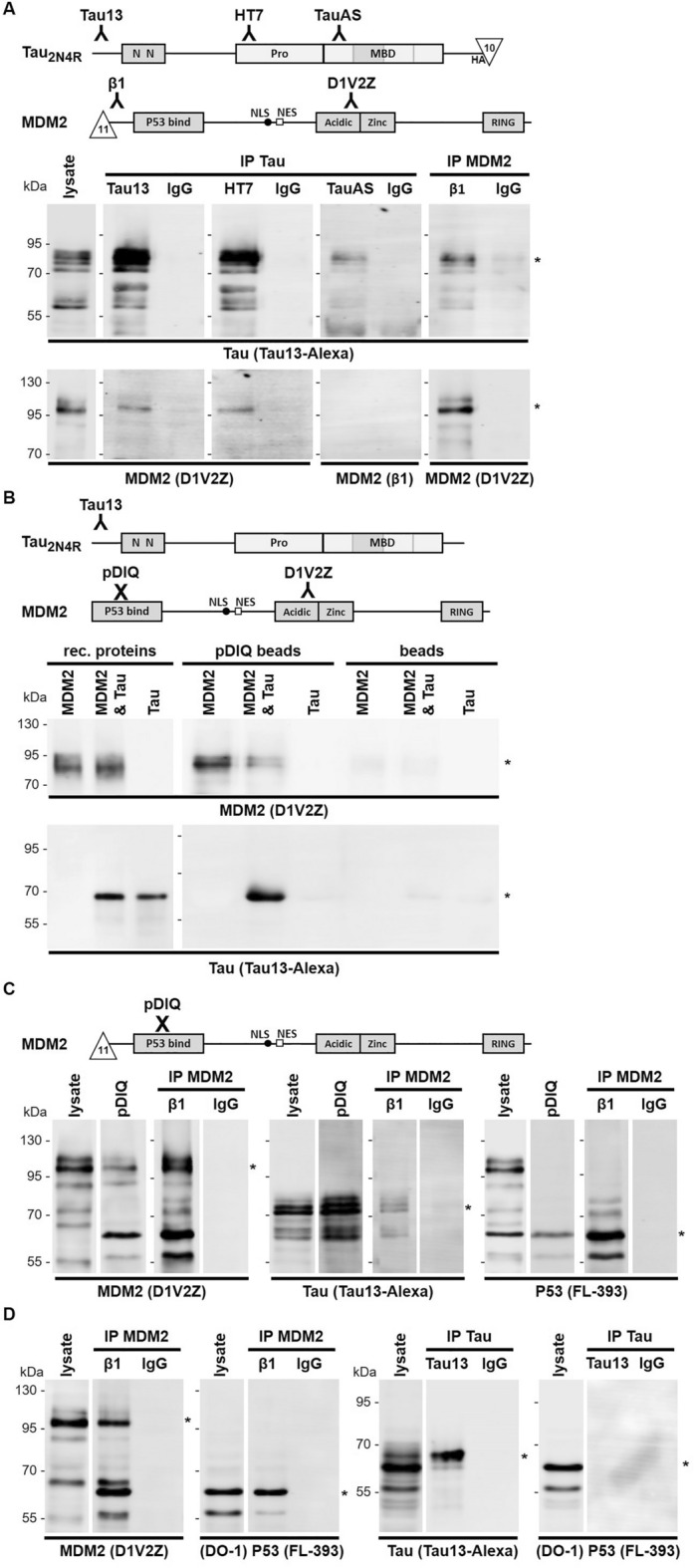
Tau interacts with MDM2. (**A**) Lysates were prepared from Hela cells with ectopic expression of Tau_2N4R_-HAT_10_ and T_11_β1-MDM2. Identical amounts of the same cell lysate were loaded directly on the gel or subjected to immune precipitation (IP) with the indicated anti-Tau antibodies or matching amounts of control IgG antibodies. A reverse immune isolation was performed with the mouse monoclonal β1 antibody against tagged MDM2 or control IgG mouse antibody. Samples were resolved on a single gel and analyzed by western blot with either the mouse Tau13-AF680 antibody, the rabbit D1V2Z antibody and anti-rabbit IgG IRDye 800CW, or monoclonal β1 and anti-mouse IgG IRDye 680RD. (**B**) The indicated purified recombinant proteins (15 ng) were loaded directly on a single gel or subjected to affinity purification on beads coupled to the pDIQ peptide or on uncoupled beads and resolved on a single gel and blotted as described. (**C**) Lysates from cells expressing T_11_β1-MDM2, Tau-HAT_10_ and T_10_HA-P53 were affinity purified on pDIQ-beads or immune purified with the β1 antibody, resolved on a single gel and blotted as described. P53 was revealed with the FL-393 antibody and anti-rabbit IgG IRDye 800CW. (**D**) Lysates from cells expressing T_11_β1-MDM2 and T_10_HA-P53 were immune purified with the β1 antibody (left). Lysates from cells expressing Tau-T_10_HA and T_10_HA-P53 were immune purified with the Tau13 antibody (right). Samples were blotted for MDM2 with the rabbit D1V2Z antibody and anti-rabbit IgG IRDye 800CW. P53 was revealed in cell lysates with the DO-1 antibody and anti-mouse IgG IRDye 680RD and after IP with the FL-393 antibody and anti-rabbit IgG IRDye 800CW. Molecular weight markers are given on the left of the blots. Original blots are presented in supplementary material (Raw WB Sola).

The binding between Tau and MDM2 was first assessed in human HeLa cells with ectopic expression of full-length human proteins. Inhibition of the proteasome was performed to stabilize MDM2 without the need of agents modifying MDM2 by e.g., antagonistic MDM2 ligands^26,27^ or induction of the DNA damage response^13^. Analysis of cell lysates revealed Tau_2N4R_ (70–80 kDa, with the C-terminal HAT_10_ epitope) and MDM2 (95–100 kDa, with the N-terminal T_11_β1 epitope) at the expected apparent molecular weight by western blot (Fig. 1A). Tau was then pulled down with antibodies against three different epitopes or with an unspecific IgG as a negative control. The amino-terminal antibody Tau13 and the central Tau antibody HT7 co-isolated Tau and MDM2 (Fig. 1A). In contrast, the antibody TauAS directed against the MBD of Tau failed to recognize the Tau-MDM2 complex, possibly indicating epitope masking when Tau is bound to MDM2 (Fig. 1A). However, TauAS isolated Tau, suggesting that the main pool of Tau was dissociated from MDM2 or that TauAS competed with MDM2 for its binding to Tau. Reversed pull down of MDM2 with the anti-tag mouse monoclonal antibody β1, but not with a control mouse IgG antibody, co-isolated Tau (Fig. 1A).

We then implemented a solid-phase affinity enrichment procedure. For this, we utilized the peptide pDIQ corresponding to an optimized α-helix of P53 displaying high affinity for the N-terminal P53 binding-pocket of MDM2^28^. We first confirmed with purified recombinant proteins, that pDIQ captured MDM2 but not Tau_2N4R_ when incubated as single proteins with the pDIQ beads, demonstrating no direct binding of Tau to pDIQ (Fig. 1B). In contrast, Tau_2N4R_ was co-isolated with MDM2 when the two proteins were incubated with the pDIQ beads; uncoupled beads served as a negative control (Fig. 1B). Starting from lysates of cells transfected for T_11_β1-MDM2, Tau-HAT_10_ and T_10_HA-P53, we then compared the isolation of MDM2 obtained by pDIQ affinity or with the anti-tag antibody. Affinity and immune isolation of MDM2, both co-isolated Tau and, as expected, its main client protein P53 (Fig. 1C). MDM2 was less efficiently captured by pDIQ than by the anti-tag β1 antibody (Fig. 1C, left panels). Perhaps the binding of pDIQ to MDM2 competed with P53 bound to MDM2 or suffered from steric hindrance. Tau co-isolated more efficiently with pDIQ-bound MDM2 (despite comparatively lower amounts of MDM2) than with immune-captured MDM2 (Fig. 1C, central panels). Much in contrast, an opposite outcome was obtained for P53 co-isolated with MDM2 when compared to Tau (Fig. 1C, right panels). Co-isolation of P53 with pDIQ-bound MDM2 was surprising, however, the binding of P53 to MDM2 involves multiple domains of the two proteins^20,21,24^. This likely explained the isolation of relatively small amounts of P53 with the pDIQ-captured MDM2 when compared to anti-tag β1 antibody-bound MDM2. P53 was also co-immune isolated with MDM2 from lysates of cells transfected with the two proteins (Fig. 1D, left panels), confirming the existence of the MDM2-P53 complex. In contrast, in cells transfected with Tau and P53, we did not find a detectable Tau-P53 complex (Fig. 1D, right panels). These data suggested that Tau and P53 may bind preferentially to distinct domains of MDM2.

**Figure 2:**
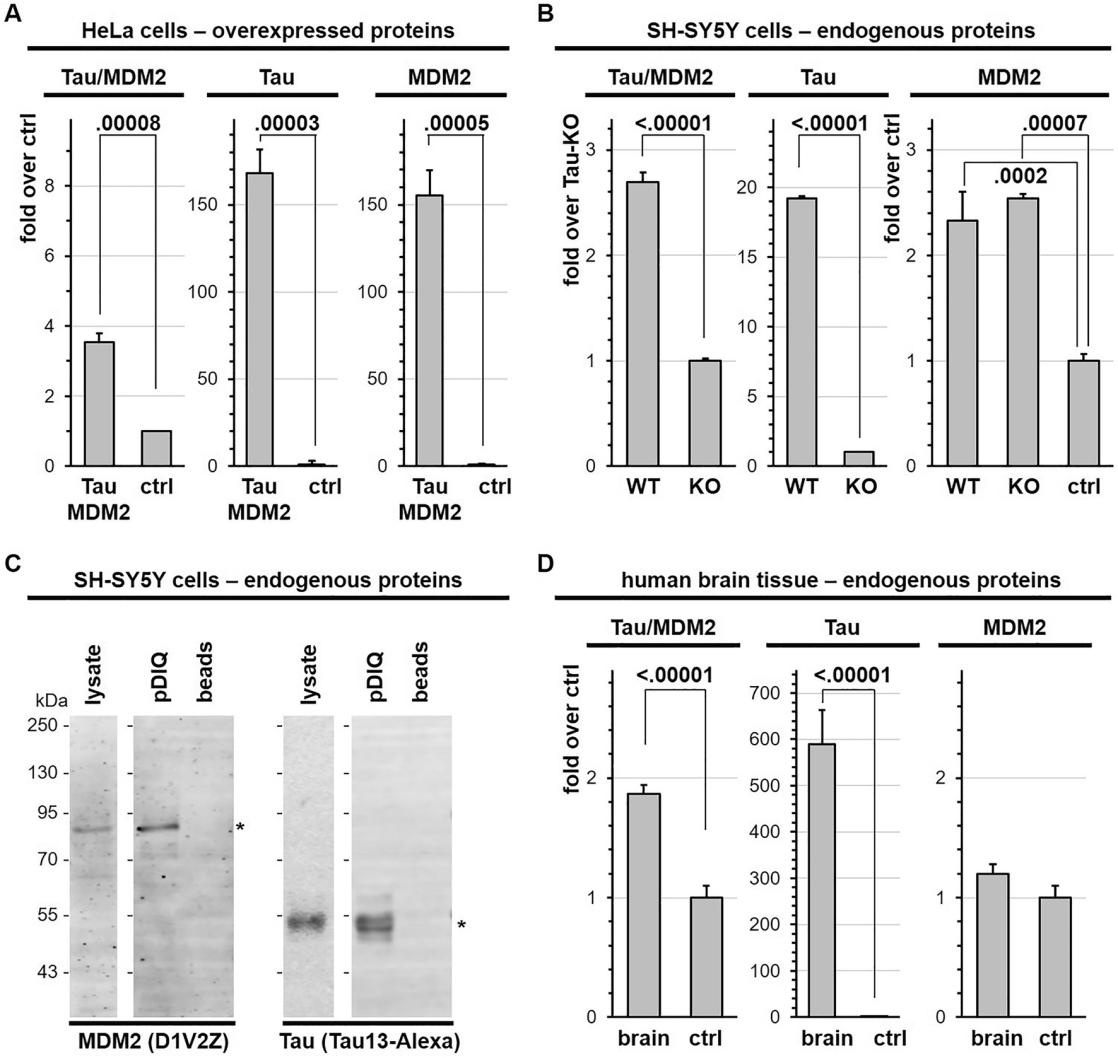
Detection of the endogenous Tau-MDM2 complex in cells and brain. (**A**) Hela cells expressing Tau_2N4R_-HAT_10_ and T_11_β1-MDM2 were treated with MG132 for 4 h before lysis and analysis by AlphaLisa to detect the Tau-MDM2 complex as well as the total amount of Tau or MDM2. Values are given as mean fold over negative control omitting one of the two primary antibodies (ctrl), n = 3, ± SD, unpaired two-tailed student t-test. (**B**) SH-SY5Y treated with MG132 were lysed and analyzed by AlphaLisa. Values are given as mean fold over negative control obtained from Tau-KO cells for Tau-MDM2 and Tau (KO) or also omitting the antibody for MDM2 (ctrl), n = 3, ± SD, unpaired two-tailed student t-test, or ordinary one-way ANOVA and Tukey’s multiple comparison test. (**C**) Identical quantities of the same SH-SY5Y cell lysate were subjected to affinity purification on the pDIQ peptide coupled to beads or on the same volume of uncoupled beads(ctrl). Cell lysates and affinity isolated proteins were resolved on a single gel and analyzed by western blot sequentially with the mouse Tau13-AF680 antibody and with the rabbit D1V2Z antibody and anti-rabbit IgG IRDye 800CW. Molecular weight markers are given on the left of the blots. Original blots are presented in supplementary material (Raw WB Sola). (**D**) Human brain homogenates from ten donors were subjected to AlphaLisa to detect the total amount of endogenous Tau-MDM2 complex as well as endogenous Tau or MDM2. Values are given as mean fold over negative control obtained by omitting one of the two primary antibodies (ctrl) n = 10, ± sem, unpaired two-tailed student t-test.

Protein–protein interactions can be detected by AlphaLisa. In this homogenous assay, antibodies against binding partners are coupled to donor and acceptor beads. Readout for bead proximity is measured by a fluorescent signal induced in the acceptor beads by short-travelling oxygen radicals released from the donor beads. Total Tau was detected with the antibody pair Tau12 (specific for amino acids 6–18 of Tau_2N4R_) and BT2 (aa 194–198). The MDM2 assay was developed in house with the antibody pair D1V2Z (surrounding Val280) and SMP14 (aa 154–167). Specificity of the signals was assessed by a negative control consisting in omitting one of the two antibodies forming the three different detection pairs. The Tau-MDM2 interaction was revealed with the antibody pair HT7 and D1V2Z in cell extracts diluted 1:250 (Fig. 2A). So, the high-sensitivity Tau-MDM2 AlphaLISA detected the complex in 0.012 µg total HeLa protein extract. In contrast, the experiments shown in Fig. 1 required the equivalent of 240 µg total HeLa protein to detect the Tau-MDM2 complex. The two single-analyte assays detected Tau or MDM2 in cell extracts diluted 1:10,000 for Tau and 1:50 for MDM2 (Fig. 2A). This high sensitivity prompted us to analyze the endogenous complex in human neuroblastoma SH-SY5Y cells. Indeed, the Tau-MDM2 AlphaLisa detected the endogenous Tau-MDM2 complex with a signal ~ 2.7-fold over the background signal measured in Tau-KO SH-SY5Y cells^13^ (Fig. 2B). The presence of endogenous Tau-MDM2 complex in SH-SY5Y was also shown upon pDIQ affinity purification (Fig. 2C). Low amounts of the Tau-MDM2 complex were also found in extracts derived from postmortem brain tissue, however the assay sensitivity appeared insufficient to detect endogenous MDM2 in total brain extracts (Fig. 2D). Acceptor beads conjugated to the anti-MDM2 antibody D1V2Z were used in the Tau-MDM2 and MDM2 assays, whereas donor beads were coupled to the anti-Tau antibody HT7 for the Tau-MDM2 complex and to the anti-MDM2 antibody SMP14 for MDM2. Thus, insufficient sensitivity for MDM2 detection was likely due to SMP14, possibly because of lower affinity when compared to HT7 or SMP14 insensitivity to its phosphorylated MDM2 epitope^29^.

### The microtubule-binding domain of Tau binds to the acidic region of MDM2

**Figure 3:**
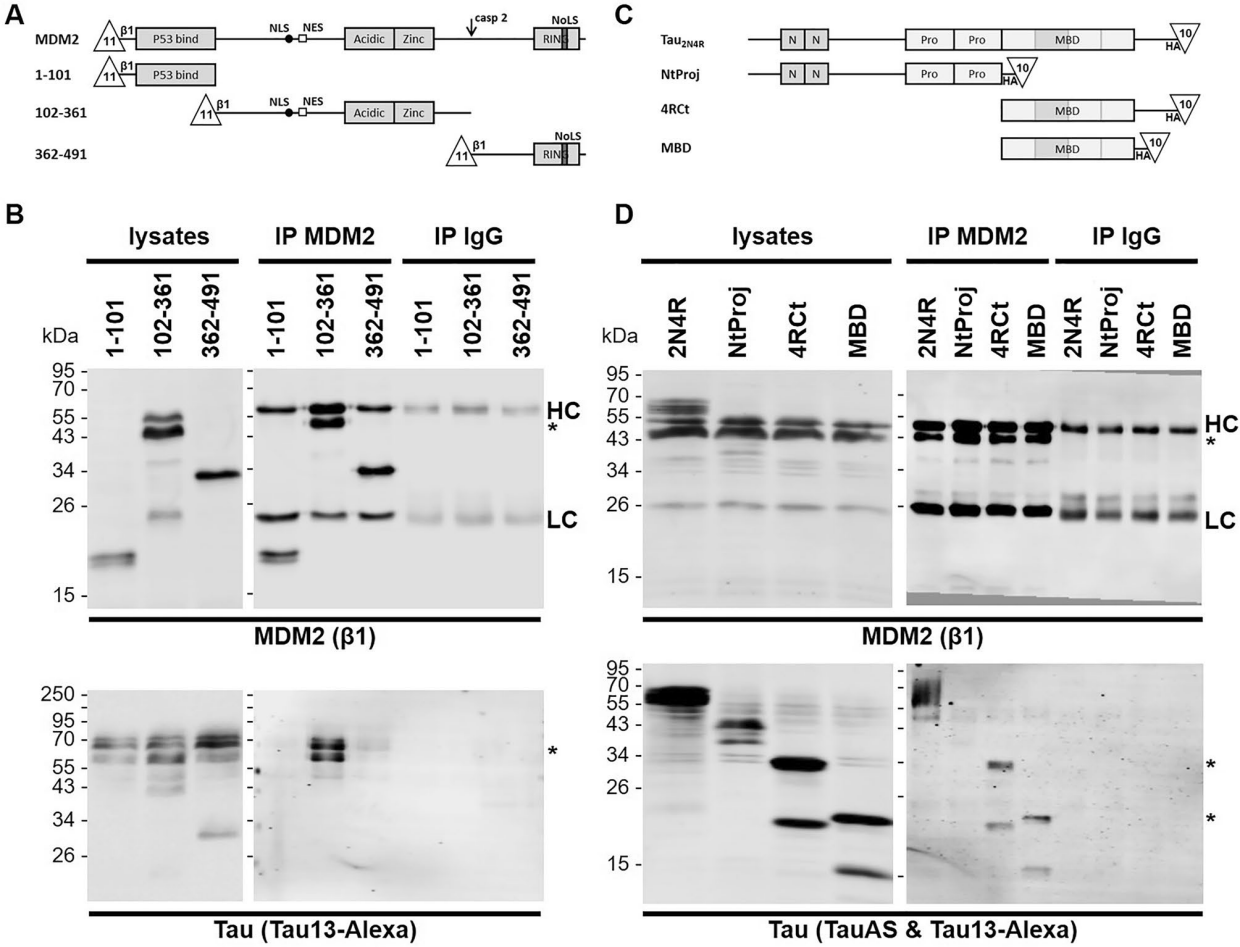
The MBD of Tau binds to the central domain of MDM2. (**A**) Schematic representation of the N-terminal T_11_β1 tagged variants of MDM2. (**B**) Hela cells co-expressing the indicated MDM2 variants and Tau_2N4R_ plasmids were treated with MG132 for 4 h before lysis. For each condition, the same quantity of cell lysate was subjected to immune precipitation (IP) with β1 (MDM2) or with matchings amounts of control mouse antibodies (IgG). All samples were resolved on the same gel and analyzed by western blot with the Tau13-AF680 antibody followed by the mouse β1 antibody and anti-mouse IgG IRDye 800CW. (**C**) Schematic representation of the C-terminal HA T_10_ tagged variants of Tau. (**D**) HeLa cells co-expressing the indicated Tau variants and T_11_β1-MDM2(102–361) were treated with MG132 for 4 h before lysis. For each condition, the same quantity of cell lysate was subjected to immune precipitation (IP) with β1 (MDM2) or with matching amounts of control mouse antibody (IgG). The samples were resolved on two gels (one for lysates and one for the immune precipitated samples) and blotted with a mixture of Tau13 and TauAS with anti-rabbit IgG IRDye 680RD followed by the mouse β1 antibody and anti-mouse IgG IRDye 800CW. Molecular weight markers are given on the left of the blots. Heavy chains (HC) and light chains (LC) of β1 are indicated. Original blots are presented in supplementary material (Raw WB Sola).

To define the interacting domains in the Tau-MDM2 complex, we expressed in HeLa cells truncated polypeptides of MDM2 or of Tau. Firstly, we co-expressed full-length Tau_2N4R_ with three complementary MDM2 fragments corresponding to the P53 binding domain (amino acids 1–101), the central region (102–361), or the remaining C-terminal polypeptide (362–491) starting from the caspase cleavage site and including the RING domain (Fig. 3A). Each T_11_β1 N-terminally tagged MDM2 fragment was immune isolated with the β1 antibody. We found that Tau was co-enriched with the central domain (102–361) but not with the other two domains of MDM2 (Fig. 3B).

We then co-expressed and immune isolated the central domain (102–361) of MDM2 from cells expressing one of the three fragments of Tau corresponding to two complementary fragments truncated between the proline-reach region and the MBD or representing only the MBD (Fig. 3C). No interaction was observed when cells expressed the N-terminal projection of Tau (NtProj, 1–243). In contrast, the C-terminal fragment of Tau (4RCt, 244–441), the MBD (244–372), and their two degradation products present in cells at similar levels than NtProj, were found to interact with the central domain of MDM2 (Fig. 3D). These data were in accord with the possible competitive binding of the MBD-specific TauAS antibody or of MDM2 to the same domain of the Tau protein (Fig. 1A).

**Figure 4:**
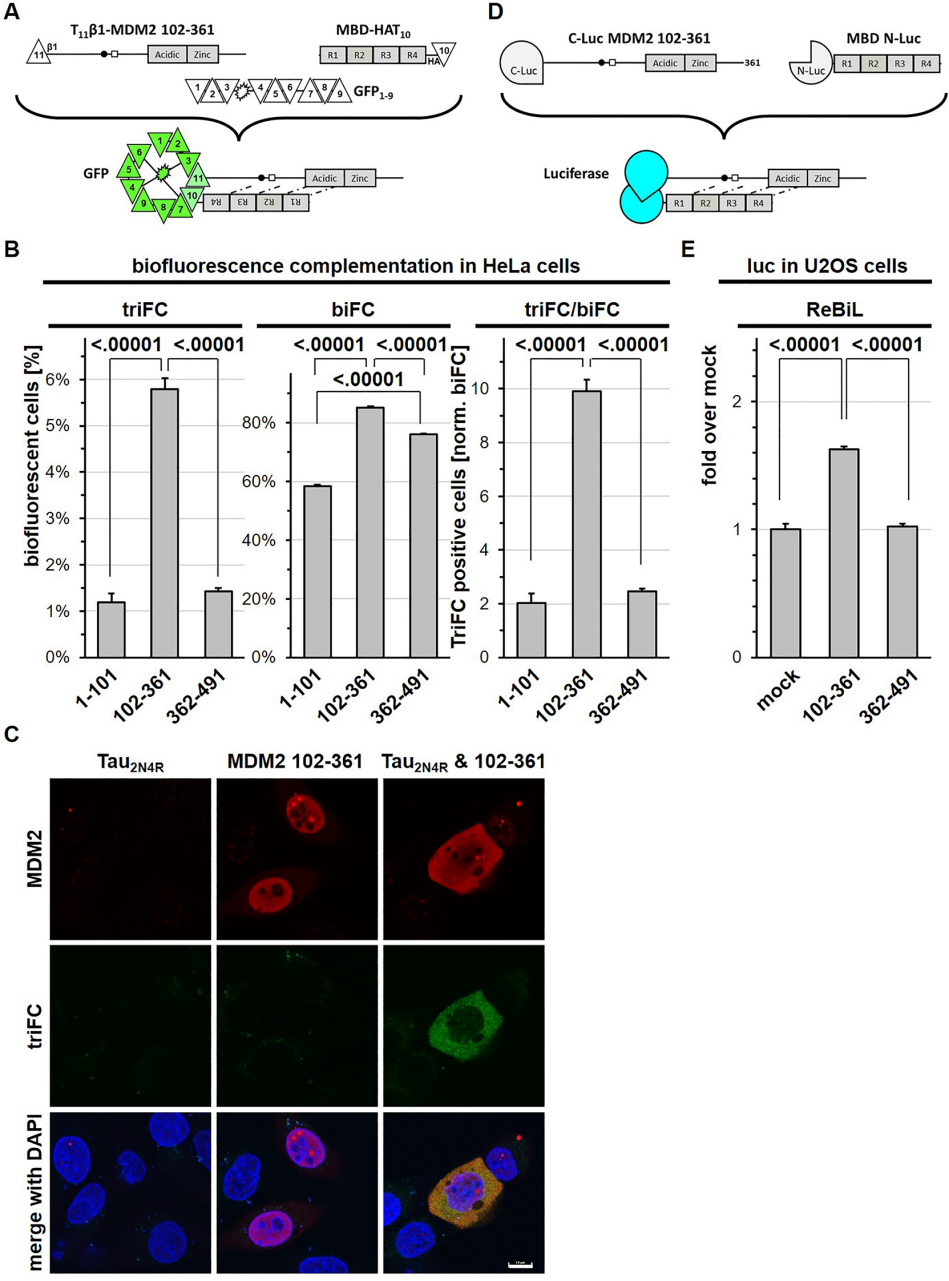
Tau-MDM2 complex detected by triFC and ReBiL. (**A**) Scheme of the principle for trimolecular GFP fluorescence complementation (triFC) to analyze the interaction between T_11_β1-MDM2 fragments and TauMBD-HAT_10_. (**B**) Hela cells co-expressing the indicated proteins were treated with MG132 and analyzed by cytofluorimetry for triFC, biFC and triFC normalized for biFC. Values are given as mean percent fluorescent cells with reconstituted GFP, n = 3, ± SD, ordinary one-way ANOVA and Tukey’s multiple comparison test. (**C**) Hela cells expressing with indicated proteins and treated with MG132 were analyzed by confocal microscopy for the ectopic expression of MDM2 (β1 antibody) or triFC signal. Merged images include nuclear counterstaining with DAPI. Calibration bar = 10 µm. (**D**) Scheme of the principle for luciferase activity reconstitution (ReBiL) to analyze the interaction between cLuc-MDM2 fragments and nLuc-TauMBD. (**E**) Lysates from U2OS cells with induced co-expression of the indicated protein fragments were analyzed for luciferase activity in the presence of luciferin. Values are given as mean fold over mock transfected cells, means of 5 measurements recorded every 3 min in technical duplicates, n = 10, ± sem, ordinary one-way ANOVA and Tukey’s multiple comparison test.

Tagging proteins with the T_10_ and T_11_ β-strands of GFP facilitate assessing protein–protein interactions in living cells by trimolecular fluorescent complementation (triFC)^30^. So, we tagged with the T_10_ the MBD of Tau and with T_11_ each one of the three MDM2 domains described above (Fig. 4A). After co-expression in cells together with the remaining GFP_1–9_ fragment, single-cell analysis by cytofluorimetry revealed whether living cells contained detectable amounts of the complex resulting from the interaction between Tau and MDM2. The data obtained confirmed the preferential interaction of the MBD with the central domain of MDM2 by an independent read-out (Fig. 4B, left panel). Bimolecular fluorescence complementation (biFC) of the MDM2 fragments tagged with T_11_β1 in the presence of GFP_1–10_ was used to determine the amount of the three MDM2 fragments present in the cells (Fig. 4B, central panel)^30^. Normalization of triFC (Tau-MDM2 complex) with biFC (MDM2)^30^ confirmed that the highest percentage of cells positive for the Tau-MDM2 complex expressed the MBD of Tau together with the central domain of MDM2 (Fig. 4B, right panel). The triFC analysis added information on the subcellular distribution of the Tau/MDM2 complex. In the absence of ectopic Tau expression, immune staining of the central domain of MDM2 in fixed HeLa cells revealed its nuclear localization, in accordance with the presence of the two nuclear localization regulatory sequences of MDM2. In contrast, co-expression of Tau enriched the triFC-positive Tau/MDM2(102–361) complex in the cytosol (Fig. 4C).

Protein reconstitution utilizing the enzymatic activity of luciferase as a read-out is an alternative, reversible, approach to analyze protein dimerization. The ReBiL assay exploits an inducible bidirectional promoter for matching transcription of the mRNAs encoding for complementing luciferase fragments^31^. We inserted in the parental plasmid the MBD of Tau down-stream of the N-terminus of luciferase and the central domain of MDM2 down-stream of the complementing luciferase portion (Fig. 4D). We then determined luciferase activity in cell extracts of transiently transfected human U2OS cells, which were used previously to analyze the P53-MDM2 complex^31^. A distinct luciferase signal was obtained for cells expressing the MBD of Tau and the central region of MDM2, whereas no signal above basal levels was detected for cells expressing the MBD with the C-terminal region of MDM2 (Fig. 4E).

### FTLD-Tau displays reduced impairment of MDM2-ubiquitination of P53 when compared to wild-type Tau

**Figure 5:**
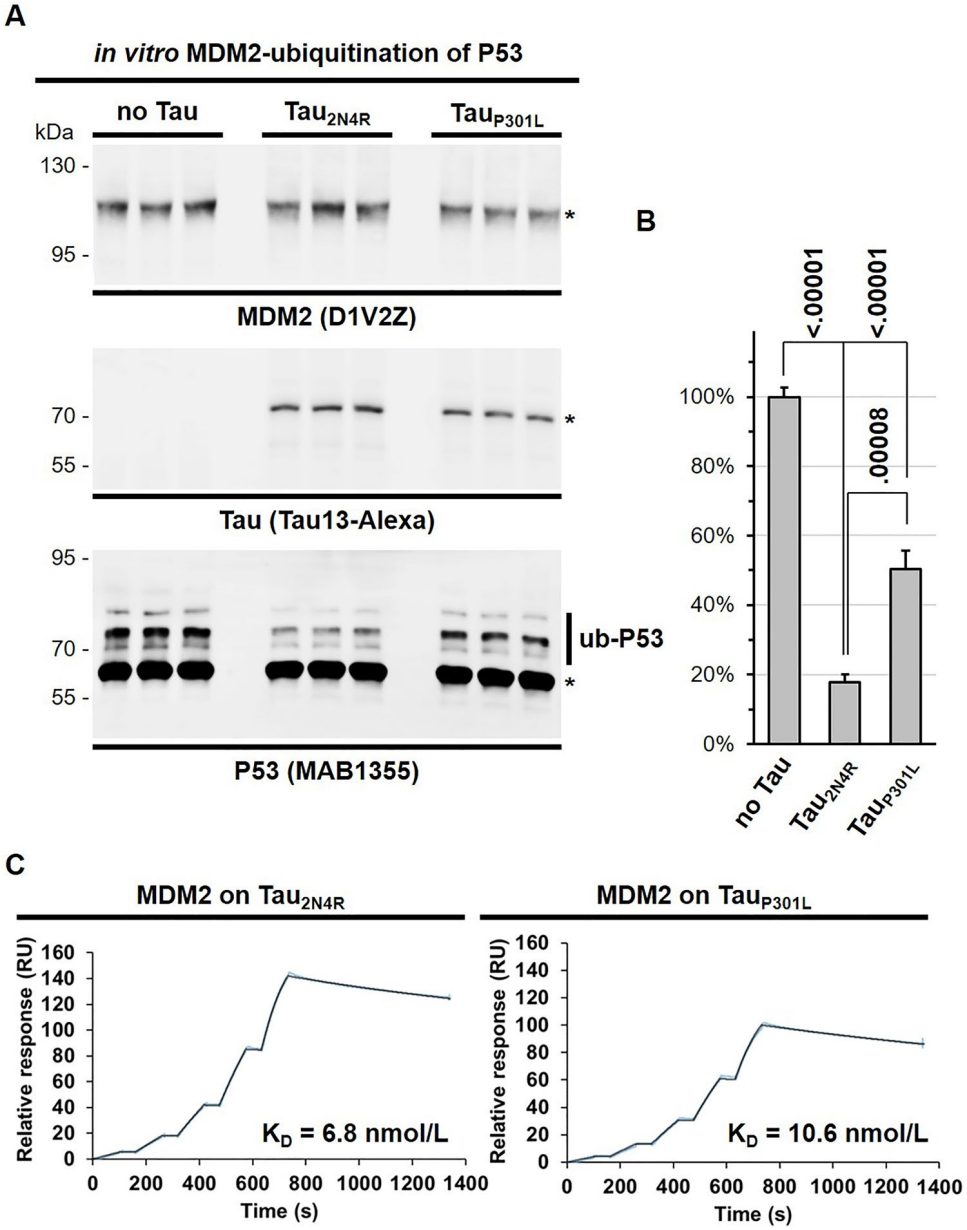
Tau inhibits MDM2-ubiquitination of P53. (**A**) In vitro P53 ubiquitination by MDM2 was performed in the absence or presence of wild-type Tau (Tau_2N4R_) or mutant Tau_2N4R_ (Tau_P301L_). Triplicate conditions of the reaction were analyzed by western blot with the indicated antibodies against MDM2, Tau or P53. Molecular weight markers are given on the left of the blots. Original blots are presented in supplementary material (Raw WB Sola). (**B**) Quantification of ubiquitinated P53 (Ub-P53) was normalized for unmodified P53 (asterisk). Values are given as mean percent of no Tau conditions, n = 3, ± SD, ordinary one-way ANOVA and Tukey’s multiple comparison test. (**C**) The affinity of the Tau-MDM2 complex was determined by SPR utilizing sensors coated with Tau_P301L_ or Tau_2N4R_ and flows containing twofold increasing concentrations of MDM2 starting from 37.5 nM. The assay was run with full-length human recombinant proteins.

The E3 ubiquitin-protein ligase activity of MDM2 is easily monitored with a commercial kit containing all required reagents necessary for the ubiquitination of P53 and analysis of apparent molecular shift by western blot. We tested whether the binding of Tau to MDM2 was reflected by the inhibition of its activity. For this, the assay was performed in the absence or presence of recombinant Tau_2N4R_ and ubiquitinated P53 was detected as slower migrating immune reactive bands with a pan-specific antibody (Fig. 5A).

As expected, MDM2 activity was reduced by ~ 80% upon addition of Tau to the reaction mixture. Strikingly, the autosomal dominant FTLD-Tau variant P301L reduced the antagonistic activity of Tau on MDM2-ubiquitination of P53 to a substantial extent (Fig. 5B). The P301L mutation of Tau is localized within a highly conserved amino acid sequence of the MBD, which in turn we showed to bind to the central region of MDM2. To determine whether the presence of the P301L mutation affected the binding to MDM2, we determined the affinity of Tau for MDM2 by surface plasmon resonance (SPR). The binding affinity of the Tau/MDM2 complex corresponded to a KD = 6.8 nmol/L for wild-type Tau_2N4R_ in contrast to Tau_P301L_ with a reduced affinity = 10.6 nmol/L (Fig. 5C). These data likely explain the reduced inhibitory potency of mutated Tau towards the MDM2-ubiquitination of P53. Consistent with these findings, mutated Tau_P301S_ was less efficiently co-immune isolated with MDM2 than Tau_2N4R_ when expressed in cells (Supplementary Fig. 1).

### Aberrant accumulation of MDM2 in AD-linked brain NFTs

**Figure 6:**
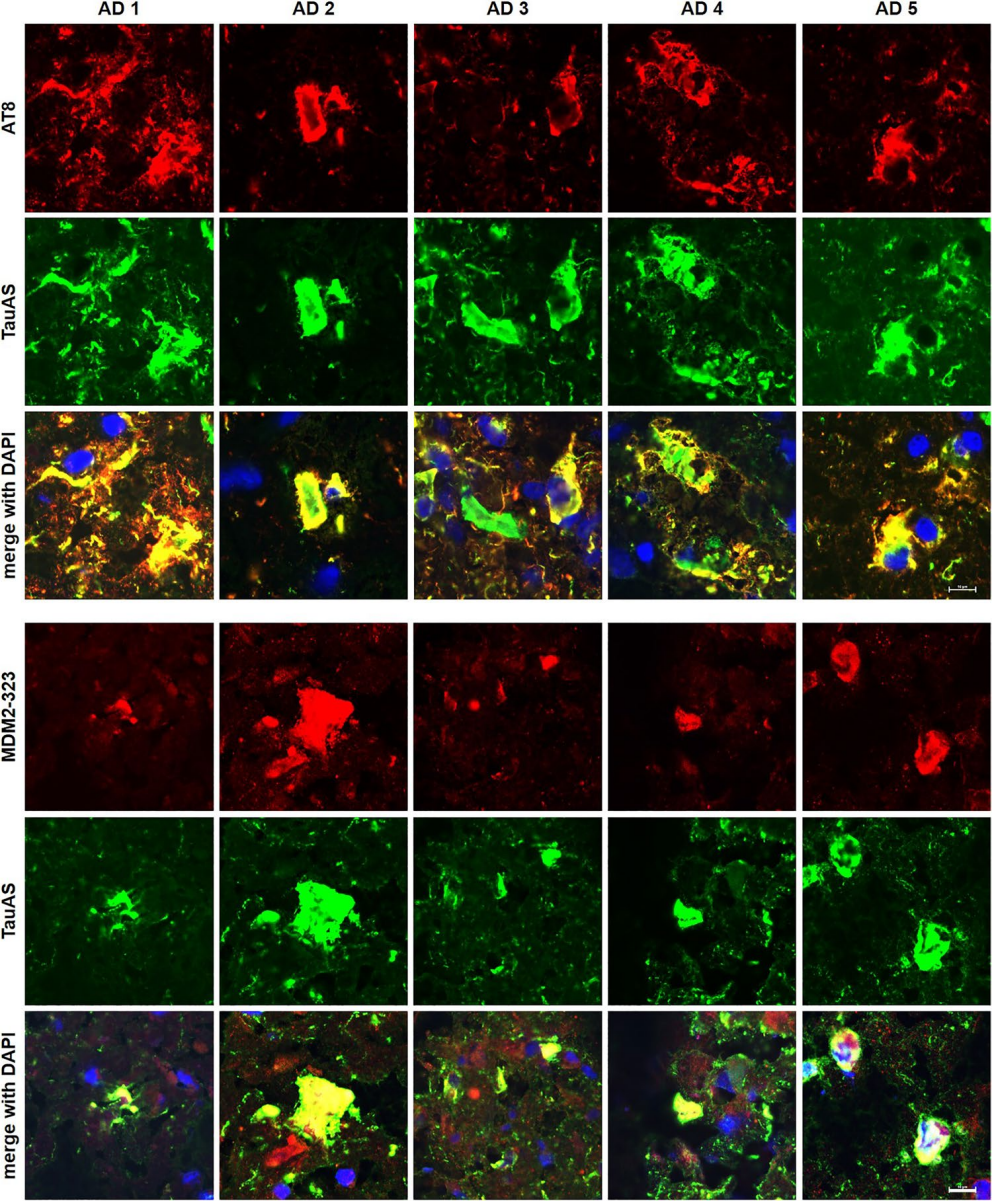
Abnormal MDM2 accumulation in Tau neurofibrillary tangles in the AD brain. Frozen frontal cortex sections from five AD brains were stained either with the mouse antibody AT8 against phosphorylated Tau and the rabbit antiserum TauAS against total Tau (upper panels) or the mouse antibody MDM2-323 against MDM2 and total Tau (lower panels). Merged images include nuclear counterstaining with DAPI. Calibration bar = 10 µm.

Prompted by the data obtained so far, we investigated whether fibrillar Tau deposition in the brain may alter MDM2 expression. We stained 5 μm frozen frontal cortex sections obtained from five AD brains with antibodies against Tau, phosphoTau or MDM2. As expected, the presence of hallmark neurofibrillary tangles and neuropil threads was revealed by the TauAS polyclonal antibody as well as by the AT8 antibody against a pathological phosphorylation of Tau^32^ (Fig. 6 upper panels). Sections derived from the same tauopathy brains revealed a double immune reactivity of Tau lesions with MDM2, similarly to what found for AT8 (Fig. 6 lower panels), but not with Aβ-peptide senile plaques (Supplementary Fig. 2).

## Discussion

Our data obtained by cross-validation with orthogonal technologies show that Tau binds to the E3 ubiquitin-ligase MDM2 in vitro, in cultured human cells and in human brain tissue. This yet unreported interaction results in the inhibition of MDM2-mediated ubiquitination of P53. In HeLa cells, viral oncoprotein E6 activates E6-AP mediates P53 degradation, whereas the MDM2-dependent pathway is inactive^33^. However, we showed that upon overexpression, MDM2 retained the ability to bind Tau and P53 in HeLa cells, similar to the observation made with cell lines and brain tissue in the absence of viral E6. Encompassing about 200 proteins, the MDM2 interactome include ribosomal proteins, transcription factors, tumor suppressors, DNA repair mediators, cell fate modulators, E2 ubiquitin-protein ligases and other regulators of cell function^34^. The large number of binding partners is likely to ensue from the scaffold nature of MDM2, which is organized in at least three structured domains linked by predominantly disordered polypeptides. This apparent promiscuity contrasts with the selective and tightly regulated functions attributed to MDM2. Perhaps, this is mirroring a model where different PTMs and conformations of MDM2 contribute to restrict the set of interacting partners within a cell^35^. Tissue-specific absence of binding partners may further limit the spectrum of MDM2 activities in differentiated cells. Then again, the preferential expression of Tau in neuronal populations of the central nervous system or in specific peripheral organs, may explain why the Tau-MDM2 complex eluded previous studies.

We demonstrated that the privileged domain interacting with Tau is the central region of MDM2. This MDM2 fragment encompasses the acidic domain (residues 243–301) and part of the zinc-finger domains (residues 290–335). It is also the region of MDM2 that cooperates with the canonical P53 binding domain to interact and induce a conformational switch in P53^36^ regulated by CKIδ phosphorylation^24^. Moreover, the intramolecular interaction between the central region and the RING domain produces a conformation of MDM2 unable to bind the proteasome. It is therefore anticipated that the binding of Tau to the central domain of MDM2 may interfere with these functions regulating P53 stability and activity. Consistent with this hypothesis, we report that Tau interferes with MDM2-dependent ubiquitination of P53 in an in vitro assay based on recombinant proteins. The inhibitory function of Tau on MDM2-dependent P53 ubiquitination is likely to represent the molecular mechanism that explains why stress-induced P53 stabilization is higher in Tau-expressing cells when compared to Tau-depleted cells, as reported by us previously^13^.

Numerous proteins bind to the central region of MDM2 and regulate its E3 ubiquitin-protein ligase activity. A prominent ligand with such a property is the tumor suppressor ARF, which also targets MDM2 to the nucleolus thereby reducing its availability to execute E3 ligase functions^37,38^. The zing finger of MDM2 interacts with factors regulating cell growth and proliferation, as well as free RNA proteins released from ribosome as a consequence of stress^39^. Whether Tau may interfere with these additional functions of MDM2 will be matter of future studies.

Further investigation is required to assess whether Tau also bind to MDM4, a close homolog of MDM2. In this contest, it is interesting that in the central nervous system MDM2 needs MDM4 to regulate P53^40^, whereas in other tissues MDM2 alone is sufficient to prevent accumulation of P53^41^. Since MDM4 lacks E3 ligase activity, binding of Tau to MDM4 may preferentially modulate P53 transcription^42^. MDM2 and MDM4 play a key role in cancer by controlling P53 activity but they are also associated with aging and senescence in a P53-independent manner. As an example, a germline mutation in MDM4 is associated to shortened telomeres and increase of senescence in a rare congenital condition^43^, and we reported increased senescence in Tau-depleted cells^13^.

The binding of Tau to MDM2 appears to require its MBD. Tau is described as an unfolded protein^6^, which attains a limited defined structure e.g., when its MBD binds to microtubules^44^. The current model describes that the net positive charge of the MBD and the rather acidic character of the microtubule surface contribute to their interaction. Consistent with this model, modification of the basic properties of MBD e.g., by phosphorylation, interferes with its binding to microtubules and may represent the initial step leading to pathological Tau self-assembly^6,45^. The MBD is also subjected to splicing with the alternative generation of the 4R and 3R isoforms of Tau, which show differential function and implication in disease^46,47^. Moreover, most autosomal dominant FTLD-Tau mutations target the MBD^48^, possibly affecting its splicing, its modification by PTMs and its ability to acquire defined structures when self-assembling. In this contest, it is intriguing that the FTLD-Tau P301L shows less affinity for MDM2, and less inhibitory potency against its E3 protein-ubiquitin ligase activity. On one hand, this finding may suggest a role for the P53/MDM2 axis in neurodegeneration. Moreover, considering the importance of P53/MDM2 axis in cancer, it is remarkable, and possibly now rational, that FTLD-Tau mutations are linked to increased cancer incidence^15^, that hyperphosphorylated and insoluble Tau is detected in some cancers^49^, and that Tau expression correlates positively or negatively with survival in various cancers^14^.

The central domain of MDM2 also contains the nuclear import/export signals important for the subcellular distribution of MDM2. Interestingly, the presence of Tau caused MDM2 mislocalization in the cytoplasm that may result by Tau-mediated cytosolic retention or by the interference with the nuclear import/export signals of MDM2 possibly masked upon Tau binding. This Tau-mediated mislocalization of MDM2 may further contribute to increased nuclear P53 stability and activity^13^. Consistent with this effect of Tau on the normal subcellular distribution of MDM2, we report aberrant MDM2 accumulation within Tau lesions of the human AD brain. The existence of brain MDM2 and its possible role in neurodegenerative disorders have not been much investigated so far. Our observation that MDM2 accumulates in neurofibrillary tangles calls for filling this gap in knowledge. However, a possible role of P53 in CNS disorders has been proposed^50^. Whereas cancers are linked to P53 inactivation, in neurodegeneration the level and activity of P53 appear increased^51^. Genetic manipulation of P53 family members in mice affects aging and cognitive decline^52,53^. Loss of nuclear localization of P53 and P53 oligomers in the brain of AD and tauopathy mouse models is linked to a defective DNA damage response^54^. Although, the contribution of MDM2 in this process was not explored. Consistent with this, increased DNA damage is found in AD^10^ and persistent DDR causes neuronal senescence and upregulation of pro-inflammatory factors^55^. Finally, abnormal P53 species are considered as potential biomarkers of AD^56^. Importantly, nuclear Tau can form a complex with P53, Pin1 and PARN, a mRNA stability regulator, including that of P53^57^. The cis/trans isomerase Pin1^58^ modifies Tau phosphorylation and is also sequestered into Tau lesions^59^ as now described for MDM2. Moreover, Pin1 affects P53 phosphorylation, conformation, and the interaction with MDM2^60^. As a consequence, Tau and Pin1 facilitate P53-mediated PARN activation and the transcription deregulation in neurodegeneration and cancer^57^. P53 also associates to Tau oligomers^54^, suggesting that Tau intervenes on the P53-MDM2 axis by convergent mechanisms depending on the cell status.

The link between Tau and the P53-MDM2 axis may likely explain the de-regulation of the P53 pathway and other P53- and/or MDM2-dependent signaling in neurodegeneration as well as the observed correlations between multiple aspects of cancer and *MAPT* expression.

## Materials and methods

### DNA Plasmids

For most experiments, the plasmid pcDNA3 drove ectopic expression of Tau, MDM2 and P53 in human cell lines. We took advantage of parental plasmids for N- or C-terminal protein tagging already available in the laboratory^30^. Ectopic proteins carry thus the tenth (T_10_ with the HA epitope) or the eleventh (T_11_ with the epitope recognized by the β1 antibody, or S_11_) β-strand of an optimized form of superfolder GFP^30,61^. Biofluorescence reconstitution was obtained by co-expression with the first nine (GFP_1–9_) or ten (GFP_1–10_) β-strands of GFP. The cDNA encoding the protein fragments (Supplementary Table 1) were obtained by the polymerase chain reaction (PCR) using human full-length templates and specific oligonucleotide primers (Supplementary Table 2).

For generating ReBiL cell lines we started from parental targeting plasmids generously provided by Dr. Leo Li and Dr. Geoffrey Wahl (Salk Institute, La Jolla, California). The plasmids carry a bidirectional tetracycline-inducible promotor (TREbi) for expression of proteins fused to N-terminal (nLuc) and C-terminal (cLuc) complementary luciferase fragments and HA-tags^31^. The plasmid pSME-501 was obtained starting from pLI635 by in frame cloning of the cDNAs encoding for the MBD of Tau and the central domain of MDM2 amplified by PCR (Supplementary Table 2). This resulted in inducible expression of nLuc-HA-MBD and cLuc-HA-MDM2 102–361 (Supplementary Table 1). A similar strategy was followed to obtain pSME-502 that drives the inducible expression of nLuc-HA-MBD and the RING domain of MDM2 (cLuc-HA-MDM2 362–491).

### Cell culture and DNA transfections

Human neuroblastoma SH-SY5Y cells (94030304, Sigma-Aldrich) and human cervical cancer HeLa cells (kindly provided by Prof. Magdalini Polymenidou, University of Zurich, Switzerland) were cultured in complete DMEM (61965–059, Gibco) supplemented with 10% fetal bovine serum (FBS, 10270106, Gibco), 1% non-essential amino acids (NEAA, 11140035, Gibco), and 1% penicillin–streptomycin (PS, 15140122, Gibco). Human osteosarcoma U2OS cell lines 134–8 HyTK8 (parental), 134–385 (inducible expression of P53 and MDM2 fragments) were kindly provided by Dr. Leo Li and Dr. Geoffrey Wahl (Salk Institute, La Jolla, California)^31^. Parental U2OS cells were maintained in DMEM with 10% FBS, 400 μg/ml G418 (11,811–031, Gibco), 10 μg/ml Ciprofloxacin (S2027, Lubio) and 200 μg/ml Hygromycin B (10843555001, Roche). U2OS ReBiL cells were maintained in the same medium with 4 μg/ml Blasticidin (A11139-03, Gibco) instead of Hygromycin B. When indicated cells were treated with the proteasome inhibitor MG132 at 10 μM (10 mM DMSO stock, M7449, Sigma-Aldrich) or with 0.1% DMSO for 4 h before analysis.

All cells were grown at 37 °C in saturated humidity and 5% CO2 and maintained in culture for less than one month. For transient plasmid transfections with jetPRIME (114–15, Polyplus) according to the instructions of the manufacturer, cells were grown overnight on plates coated with poly-D-lysine (P6407, Sigma-Aldrich). For U2OS ReBiL lines, parental 134–8 HyTK8 cells at 80% confluency in a 6-well plate were transfected with ReBiL targeting plasmids and a cre-recombinase plasmid (pOG231) at a 2:1 ratio using jetPRIME. Transfected cells were then replated at clonogenic density in a 10 cm plate and selected with a transient treatment with 2 μM Ganciclovir (G2536, Sigma-Aldrich)^31^. Mixed populations were maintained in the presence of 4 μg/ml Blasticidin and 1 ng/ml Doxycicline (D9891, Sigma-Aldrich).

### Immune isolation and western blot

For western blots, cells cultured in 6-well plates were rinsed with PBS and total cell extracts were prepared in 50 μL of SDS-PAGE sample buffer (1.5% SDS, 8.3% glycerol, 0.005% bromophenol blue, 1.6% β-mercaptoethanol and 62.5 mM Tris pH 6.8) and denatured for 10 min at 100 °C. For gel electrophoresis, 40 μg protein was loaded on each lane. For immune isolation, cells were lysed on ice in 100 μL AlphaLisa Lysis Buffer (AL003, PerkinElmer) supplemented with protease and phosphatase inhibitor cocktails (S8820 and 04906845001, Sigma-Aldrich). Cell lysates were treated with Benzonase (707463, Novagen) for 15 min at 37 °C, centrifuged at 20,000 g for 10 min at 4 °C, the supernatants collected and diluted in HiBlock (10205589, PerkinElmer). The samples were then incubated overnight at 4 °C with 0.5 μg of the following primary antibodies: Tau13 (sc-21796, Santa Cruz), HT7 (MN1000, Invitrogen), TauAS rabbit antiserum (ab64193, Abcam) or β1 antibody^62^. Protein G-Sepharose beads (101241, Invitrogen) were added for 1 h at room temperature and washed three times in PBS with 0.1%Tween-20. Bead-bound proteins were eluted in SDS-PAGE sample buffer by boiling for 10 min at 100 °C.

After 10% SDS PAGE, PVDF membranes with transferred proteins were incubated with the primary antibodies indicated in the figures: 0.2 μg/mL Tau13-AlexaFluor (sc-21796 AF680, Santa Cruz), 0.2 μg/mL TauAS rabbit antiserum (ab64193, Abcam), 0.1 μg/mL rabbit monoclonal D1V2Z (86934, Cell Signaling), 0.1 μg/mL FL-393 rabbit antiserum, 0.4 μg/mL DO-1 antibody (sc-126, Santa Cruz) or 2.3 μg/mL β1 antibody. Primary antibodies were revealed with anti-mouse IgG coupled to IRDye 680RD or anti-rabbit IgG coupled to IRDye 800CW (Licor Biosciences, 926–68070 and 926–32211) on a dual infrared imaging scanner (Licor Biosciences, Odyssey CLx 9140). Immune reactive bands were quantified with the software provided (Licor Biosciences, Image Studio V5.0.21, 9140–500).

### Affinity purification

Cell lysates prepared as described for the immune isolation were incubated 30 min at room temperature with 5 μl conjugated beads. The synthetic 12-mer optimized pDIQ peptide (ETFEHWWSQLLSGGC, customer synthesis by Genscript) matches the amino-terminal α-helix of P53 and displays higher affinity for MDM2 than P53 by fitting in a deep pocket at the amino-terminal P53-binding domain of MDM2 (IC_50_ 8 nM^28^;) as supported by the X-ray diffraction-resolved structure of the pDIQ-MDM2 complex^28^ (https://www.ebi.ac.uk/pdbe/entry/pdb/3jzs/) and by molecular modeling^63^. pDIQ was covalently bound to beads following the instruction of the manufacturer (L00404, Genscript). Bead-bound proteins were then analyzed as described for the immune isolation.

### AlphaLISA

Frozen frontal cortex samples were obtained from completely anonymized donors through The Netherlands Brain Bank, Netherlands Institute for Neuroscience, Amsterdam (www.brainbank.nl). All donors signed a written informed consent for brain autopsy and the use of the material and clinical information for research purposes. Local guidelines foresee that work with completely anonymized human tissue samples, as it is the case herein, does not require authorization from the local ethic committee.

About 400 mg of brain tissue was homogenized on ice by eight strokes with a glass Dounce homogenizer in approximatively 1.5 mL (~ 4 × volume/mass) of homogenization buffer containing 50 mM Tris HCl pH 7.4, 8.5% sucrose, 0.23 mM EDTA, and protease and phosphatase inhibitor cocktails. Total protein concentration was determined using the Pierce BCA protein assay kit (23227, ThermoFisher Scientific). Cell extracts were obtained as described above for the immune isolation. The concentration of all samples (brain or cell extracts) was adjusted in AlphaLisa Lysis Buffer with the dilutions indicated in the figures.

Tau protein was determined by AlphaLISA (Tau-AL271C, PerkinElmer) following the instructions of the manufacturer. A home-made assay was developed for MDM2, using D1V2Z-CaptS (D1V2Z coupled to CaptSure following the instructions of the manufacturer, 6300007, Expedeon) and CaptSure-conjugated acceptor beads (ALSU-ACAB, PerkinElmer), paired to biotinylated SMP14 (sc-965B, SantaCruz) and streptavidin-conjugated donor beads (6760002, PerkinElmer). Similarly, the Tau-MDM2 complex was detected with D1V2Z-CaptS and biotinylated HT7 (MN1000B, ThermoFisher). The assays were performed in 384-well plates (6005350, PerkinElmer) by incubating for 1 h at room temperature in the dark 10 μL cell extracts and 5 μL of a mix containing biotinylated antibody (1 nM final concentration), antibody-CaptS bound to acceptor beads (10 μg/mL final concentration) in HiBlock; followed by the addition of 5 μL of donor beads (20 μg/mL final concentration) in HiBlock for another 1 h incubation. Measurement was done with a multi-plate reader (Victor Nivo, PerkinElmer) and data were analyzed using the provided software (Victor Nivo, PerkinElmer) with excitation time of 50 ms and 575/110 nm emission time of 700 ms.

### Immune staining of human brain tissue

Frozen brain samples (see AlphaLISA method) were cut in 5 μm sections using a cryostat (Cryostar NX50). Sections were fixed for 15 min in 100% methanol at -20 °C. After 1 h in PBS containing 5% normal goat serum (16210064, ThermoFisher Scientific) and 0.3% Triton X-100 (X100, Sigma-Aldrich), the sections were stained overnight at 4 °C with primary antibodies 0.2 μg/mL TauAS, 0.4 μg/mL AT8 (MN1020, ThermoFisher) or 0.4 μg/mL MDM2-323. Secondary antibodies were incubated for 1 h at room temperature at 2 μg/mL anti-mouse IgG-Alexa594 (A-11032, ThermoFisher Scientific) or anti-rabbit IgG-Alexa488 (A-11034, ThermoFisher Scientific). Nuclei were counterstained with 0.5 μg/mL DAPI (D9542, Sigma-Aldrich) for 5 min at room temperature. Images were acquired on a fluorescent laser confocal microscope (Nikon C2 microscope).

### BiFC and TriFC analysis

GFP biofluorescence reconstitution was analyzed on cells co-transfected with the plasmids encoding for proteins tagged with the T_11_β1-tag and GFP_1–10_ (biFC), or with plasmids for proteins tagged with T_10_HA, T_11_β1 and GFP_1–9_ (triFC). Immune staining^13^ was performed on cells after fixation for 15 min at room temperature with one volume of 8% PFA (F1635, Sigma-Aldrich) in PBS. After three washes with 100 mM glycin in PBS and one wash in PBS, fixed cells were blocked for 30 min in 300 μL/well PBS containing 5% normal goat serum and 0.3% Triton X-100. Ectopic MDM2 expression was revealed sequentially with 2.3 μg/mL tag β1 antibody and 2 μg/mL anti-mouse IgG-Alexa594, both antibodies were incubated for 1 h at room temperature. Images were acquired on a fluorescent laser confocal microscope.

For cytofluorimetric determinations, transfected cells were collected by a trypsin treatment and cautiously resuspended in culture medium to obtain a single-cell suspension. Cells were then washed with ice-cold PBS, resuspended in 0.5 mL ice-cold PBS and kept on ice until analysis. 10,000 cells were analysed on an analytical device (Beckman, cytoFLEX) using the 488 nm excitation laser and the FL1 emission channel (525/40 nm). Values collected included total cell number, gated cell number and geometric mean fluorescence.

### Recombinant bimolecular luciferase complementation (ReBiL)

For the ReBiL assay cells were plated at 8500 cells/well in a 96-well plate. After induction with 500 ng/mL Doxycycline for 48 h, cells were washed with PBS and lysed in 100 mM Tris pH 7.5, 0.5 mM EDTA, 150 mM NaCl, 0.1% Triton X-100, 1 mM sodium orthovanadate, 50 mM sodium fluoride, and protease inhibitor cocktail. Cell extracts were obtained by centrifugation at 13,000 g for 5 min at 4 °C and diluted with DMEM. 50 μL/well of diluted cell extract was pipetted into a 96-well plate containing 1 volume of D-luciferin and immediately analyzed in the luminometer with 0.5 s integration time every 3 min for 5 times at 26 °C.

### P53 ubiquitination assay

MDM2-dependent ubiquitination of P53 was analyzed according to the instructions of the manufacturer (K-200B, BostonChem, BioTechne). When indicated the reaction was performed in the presence of 100 ng of recombinant human Tau_2N4R_ protein produced in *E.coli* (wild-type or Pro301Leu, kindly provided by Prof. Luca Colnaghi, Università Vita-Salute San Raffaele, Milan). The reaction was initiated by the addition of the ubiquitin-containing solution, run for 30 min at 37 °C, and terminated with the addition of 5X SDS-PAGE sample buffer and 5 min at 90 °C. Protein was run on a low percentage acrylamide SDS-PAGE and transferred protein incubated with 0.25 μg/mL P53 MAB1355 antibody provided in the kit. In addition, MDM2 and Tau were detected with 0.1 μg/mL rabbit antiserum D1V2Z and 0.2 μg/mL TauAS rabbit antiserum. Immune-positive bands were detected with IRDye-coupled secondary antibodies on a dual infrared imaging scanner. P53 ubiquitination was determined according to the instructions of the manufacturer by calculating the ratio between the ub-P53 protein bands and the unmodified P53 band (labelled with an asterisk in Fig. 5A) for each condition.

### Affinity determination by SPR

The affinity between recombinant human MDM2 (E3-204-050, Biotechne) and recombinant human Tau_2N4R_ (wild-type or Pro301Leu) was measured at 25 °C by Surface Plasmon Resonance (SPR). Buffer exchange was necessary for MDM2. For this, desalting columns were employed following the instructions of the manufacturer (89882, ThermoFisher Scientific). The running buffer for MDM2 was 10 mM HEPES pH 7.4, 150 mM NaCl, 3 mM EDTA and 0.005% Tween-20. Both Tau variants were immobilized on a CM5 chip (Cytiva) through standard amine coupling and five twofold increasing concentrations of MDM2 (starting from 37.5 nM) were injected using a single-cycle kinetics setting. The data were corrected for unspecific binding and solvent effect, and curve fitting was performed with the Biacore Insight Evaluation Software (Cytiva). The fitting passed the quality control check included into the software. The measures were performed with a Biacore 8 K instrument (Cytiva).

### Statistics and reproducibility

Statistical analysis was performed with GraphPad Prism version 8.4 using the method specified in the legend of each figure. Exact p-values are specified in the figures. All quantifications were performed based on at least three independent biological replicates. Sample size, number of replicates and how they are defined is specified in the figure legends. When indicated, western blots and microscopic images are shown as representative data.

## Supplementary Information

Supplementary Figures.

Supplementary Figures.

Supplementary Figures.

Supplementary Tables.

## Data Availability

All data generated or analyzed during this study are included in this published article and its supplementary information files.
