# Supplementary material for: Tau protein binds to the P53 E3 ubiquitin ligase MDM2
Source: Sci Rep. 2023 Jun 23;13:10208. doi: 10.1038/s41598-023-37046-8 (PMC10290082; doi:10.1038/s41598-023-37046-8)

FIGURE 1A raw data (Comassie blue staining)

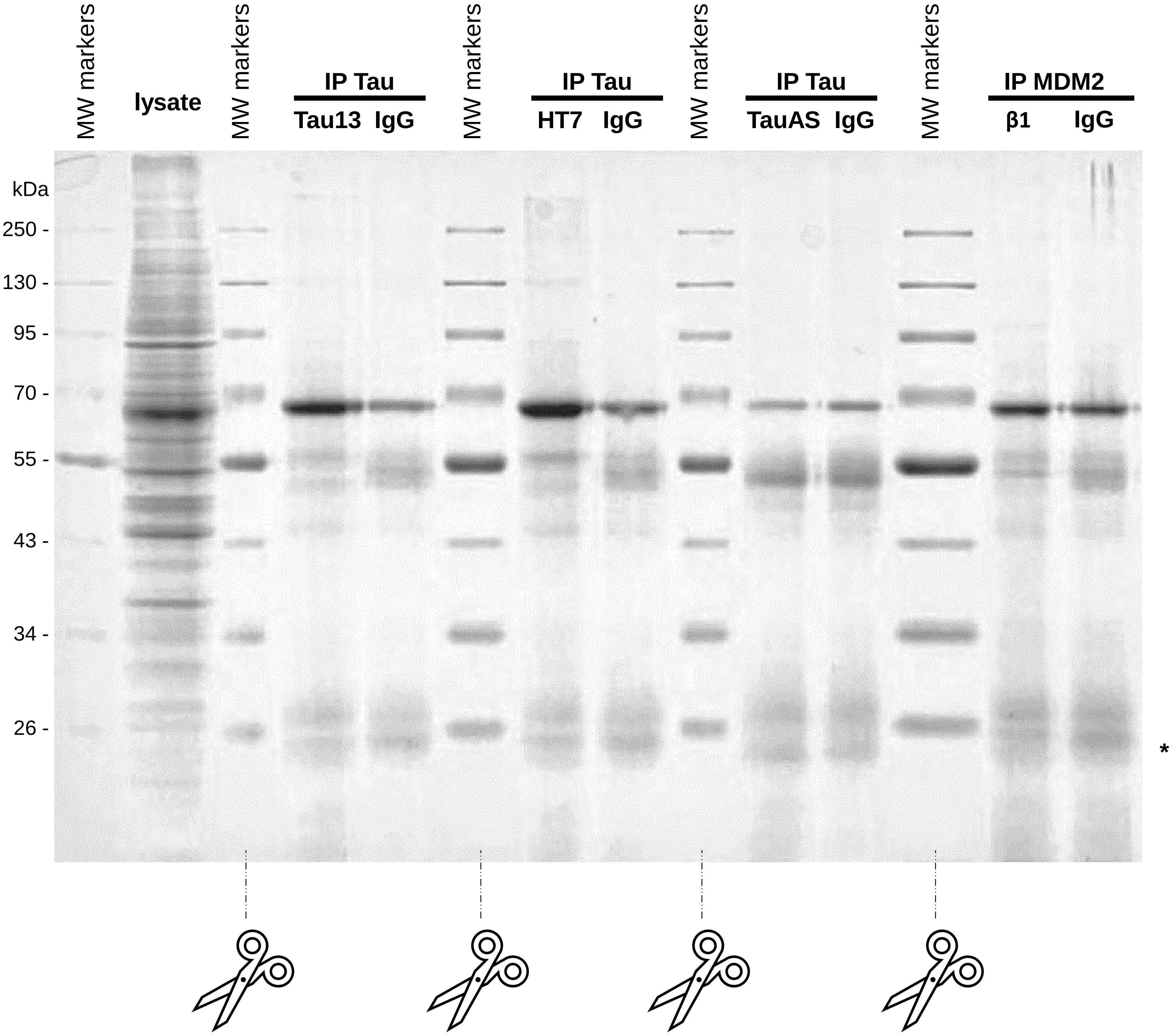

FIGURE 1A raw data Tau WB (680 nm emission)

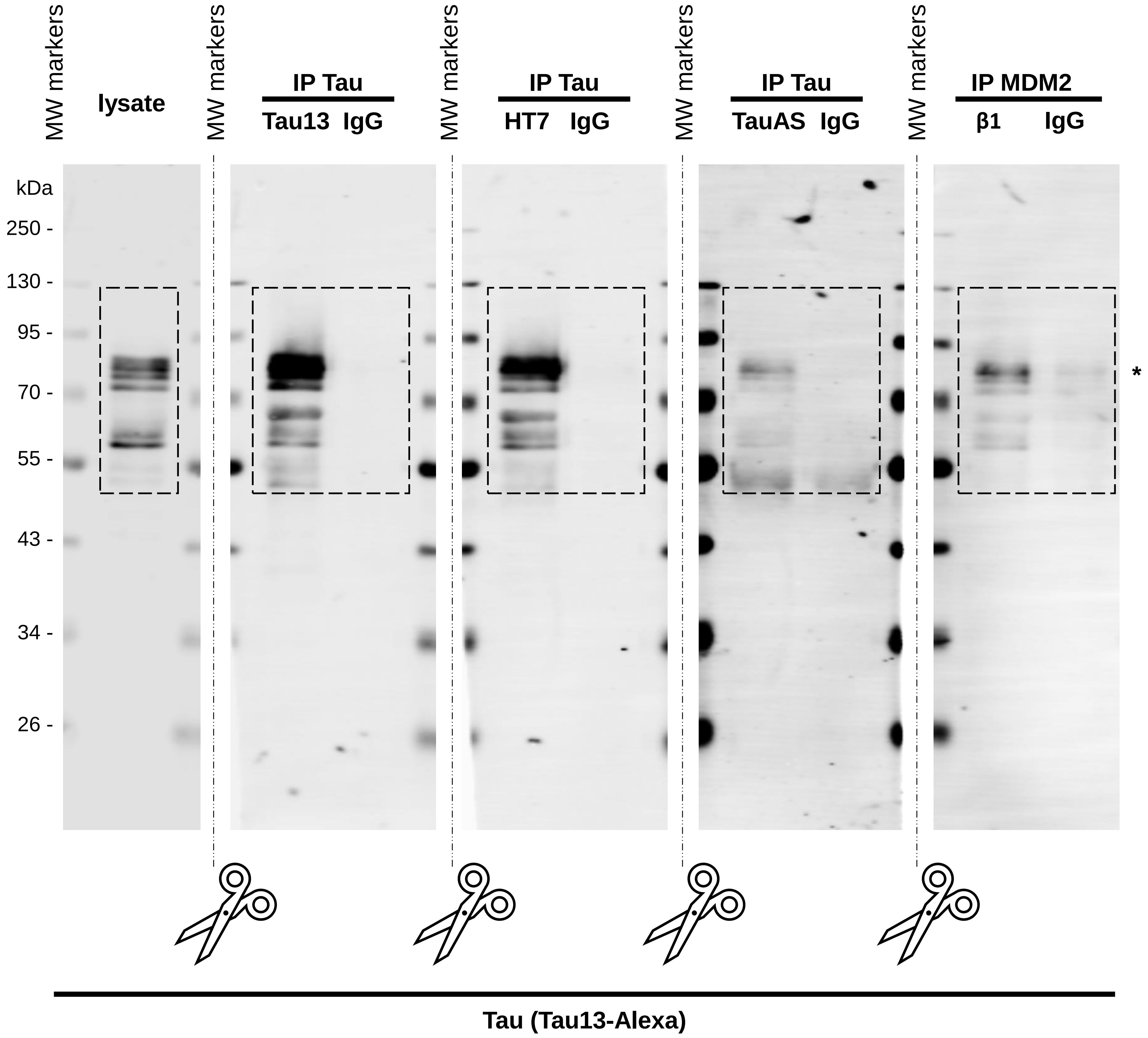

FIGURE 1A raw data MDM2 WB

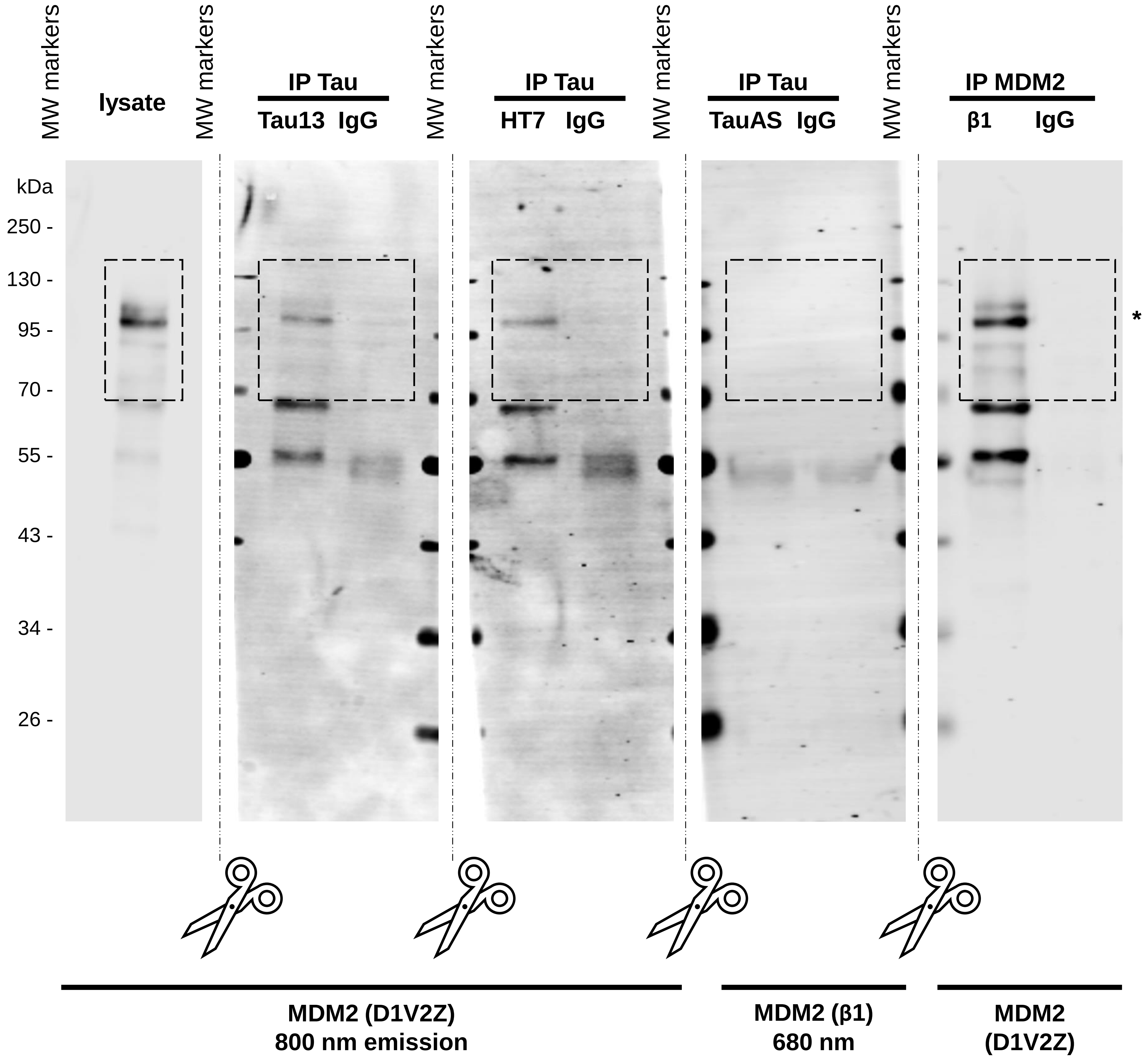

FIGURE 1B raw data affinity purification, MDM2 WB (800 nm)

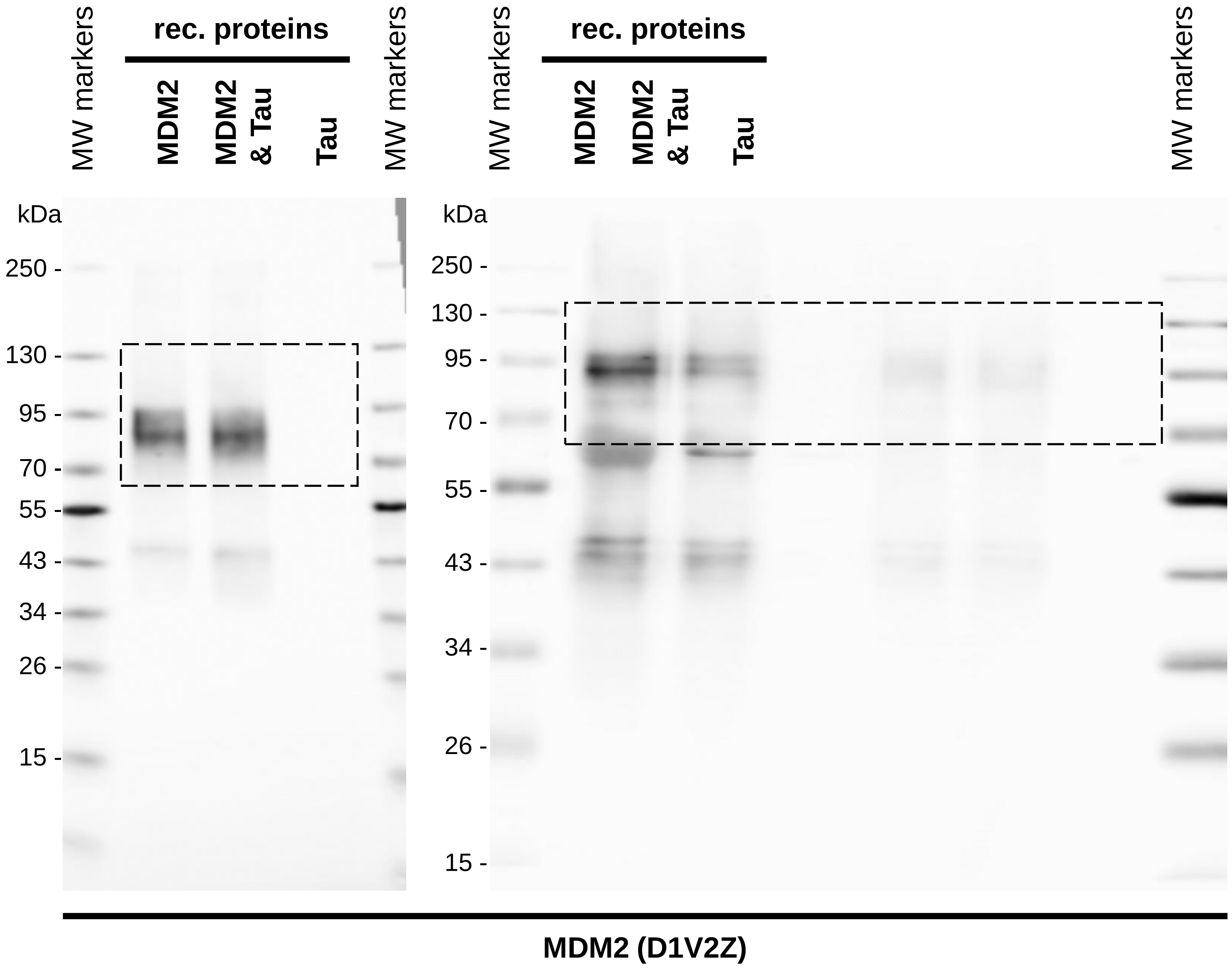

FIGURE 1B raw data affinity purification, Tau WB (680 nm)

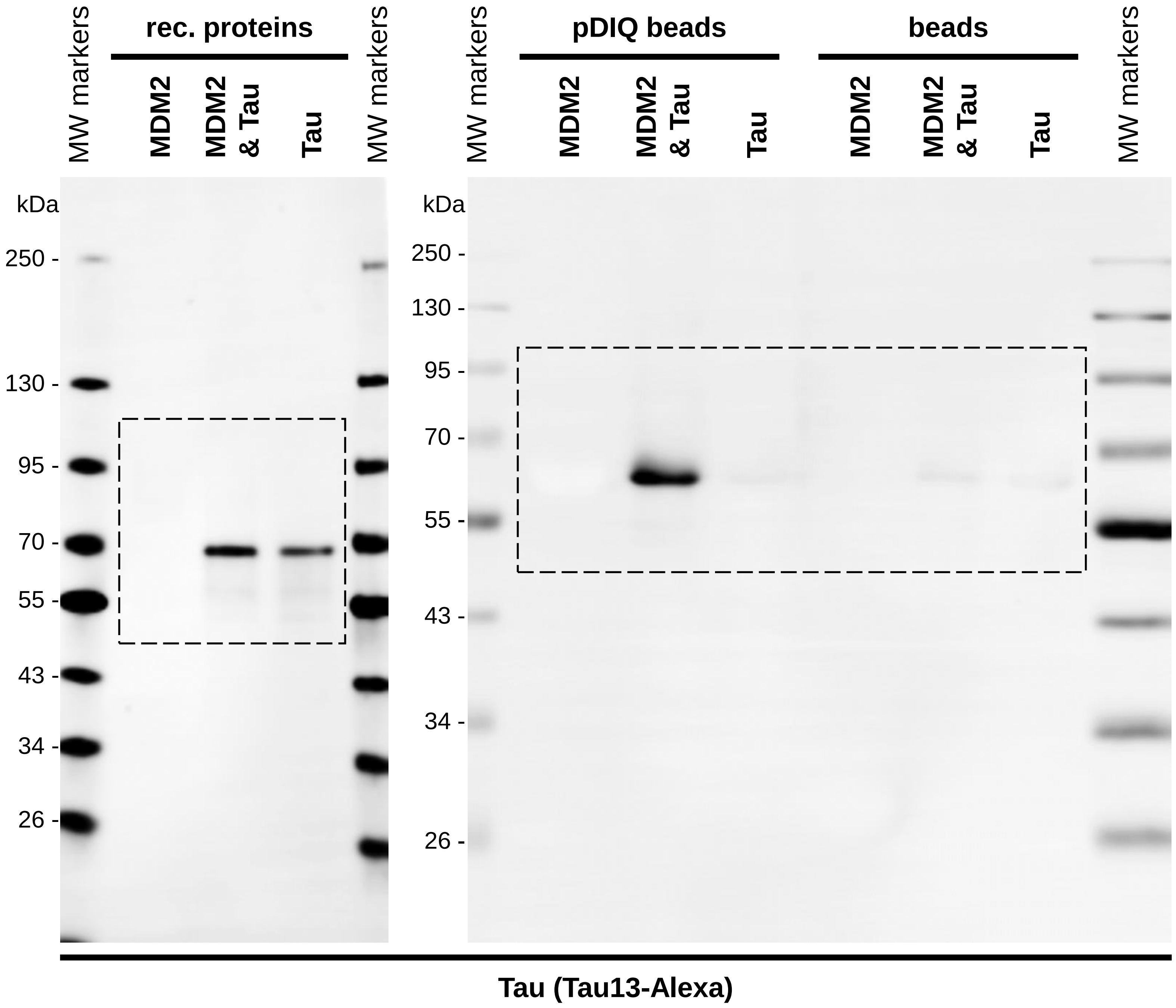

FIGURE 1C raw data MDM2 WB (800 nm emission)

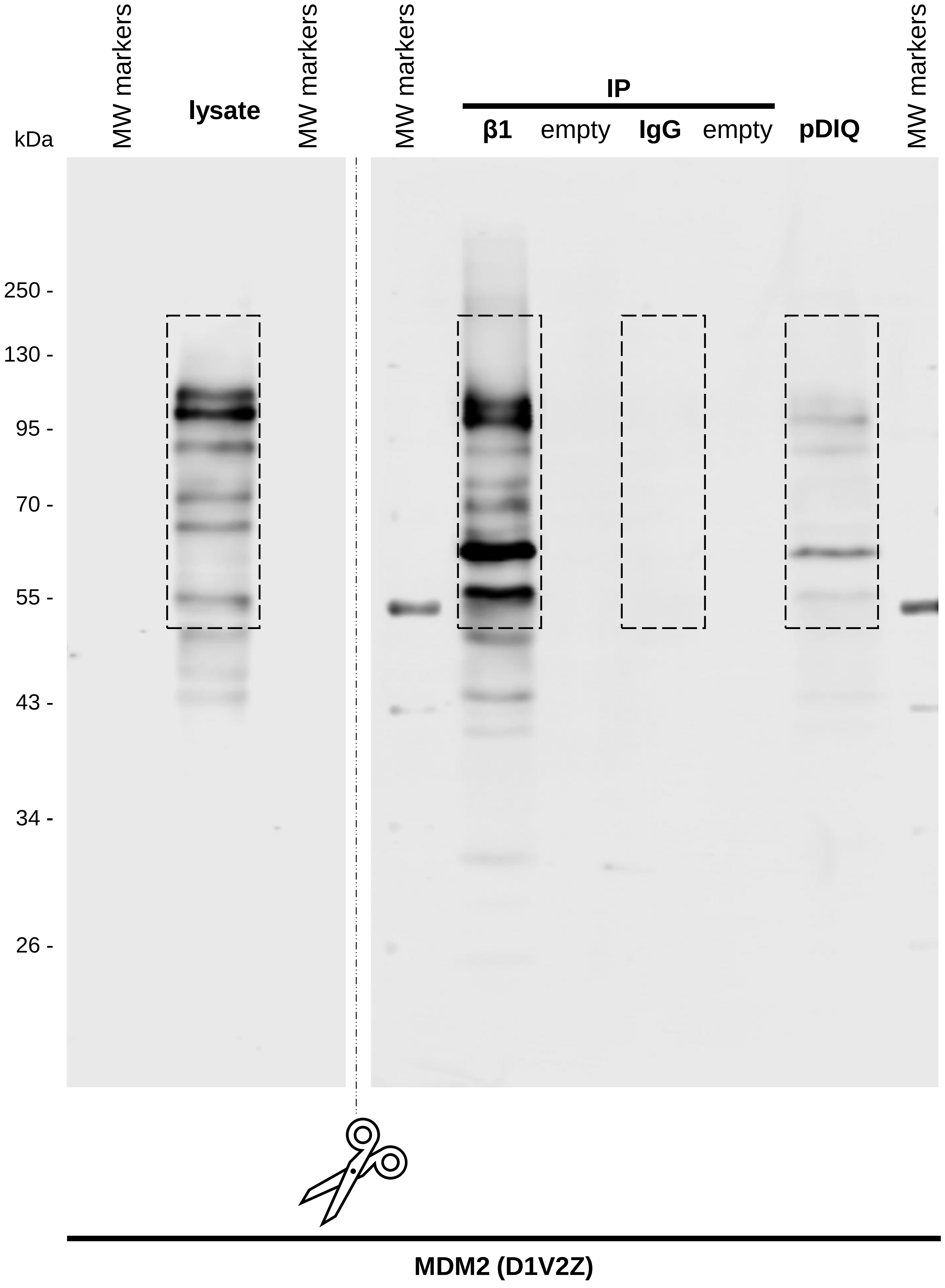

FIGURE 1C raw data Tau WB (680 nm emission)

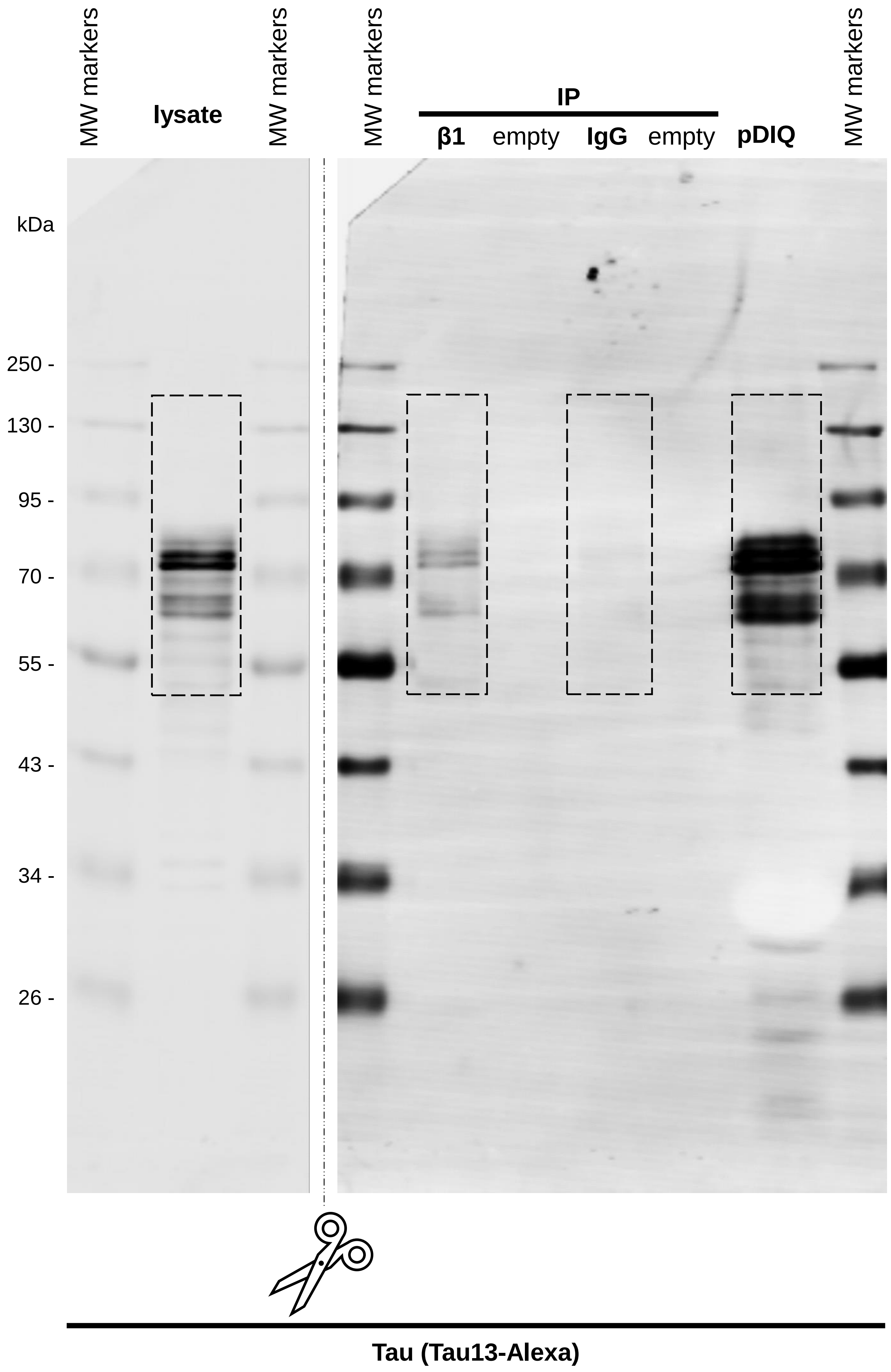

FIGURE 1C raw data P53 WB (800 nm emission)

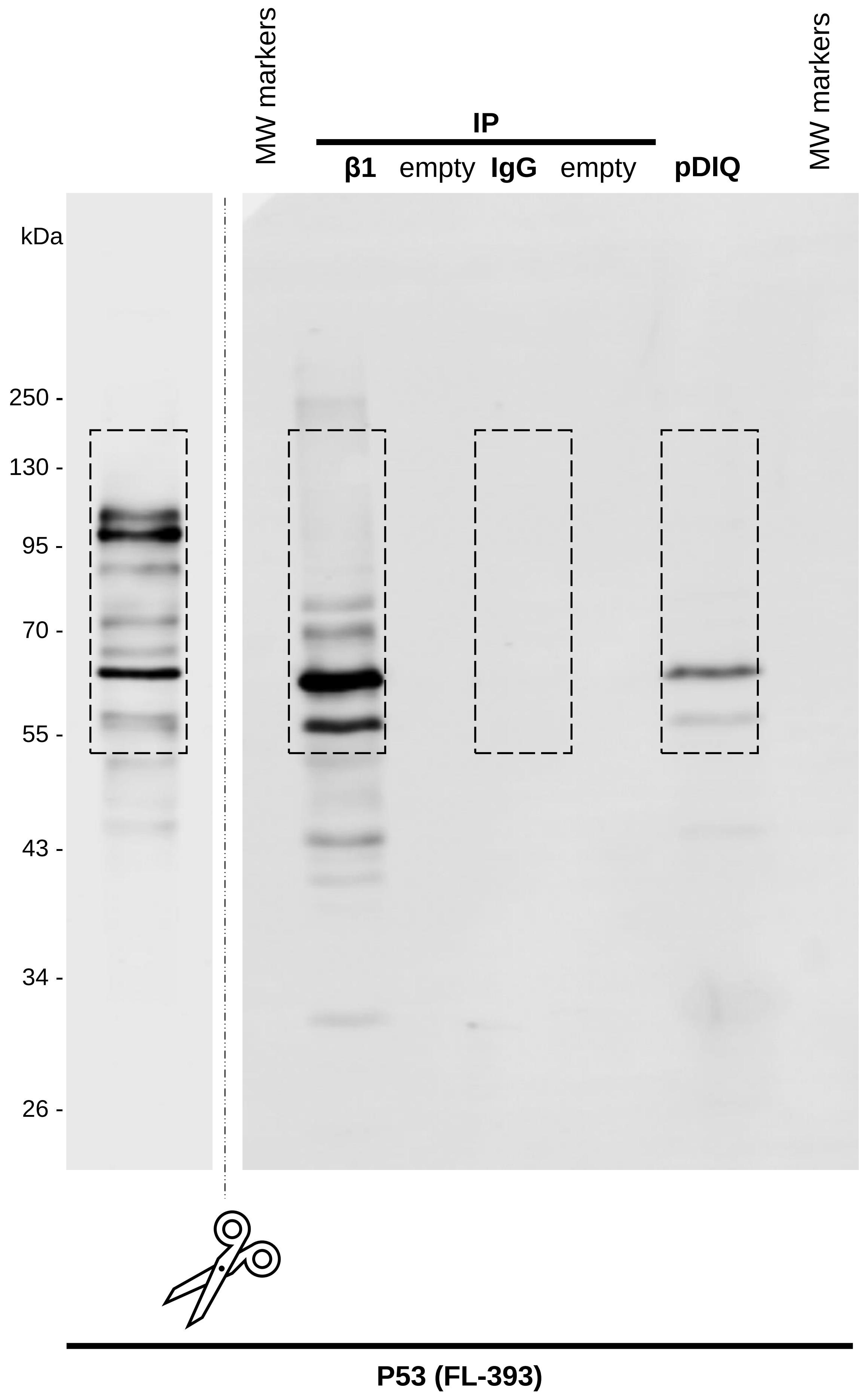

FIGURE 1D raw data IP MDM2

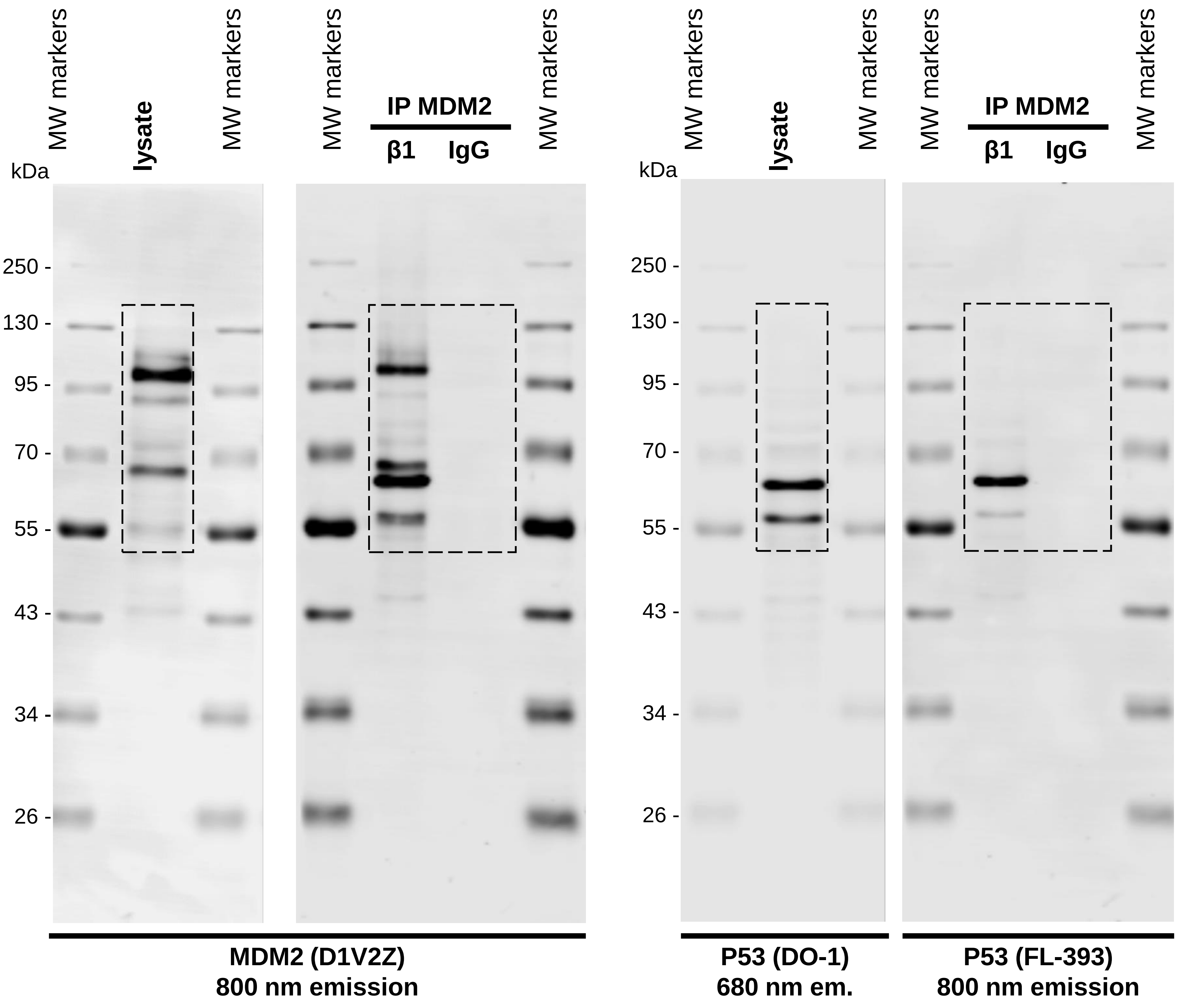

FIGURE 1D raw data IP Tau

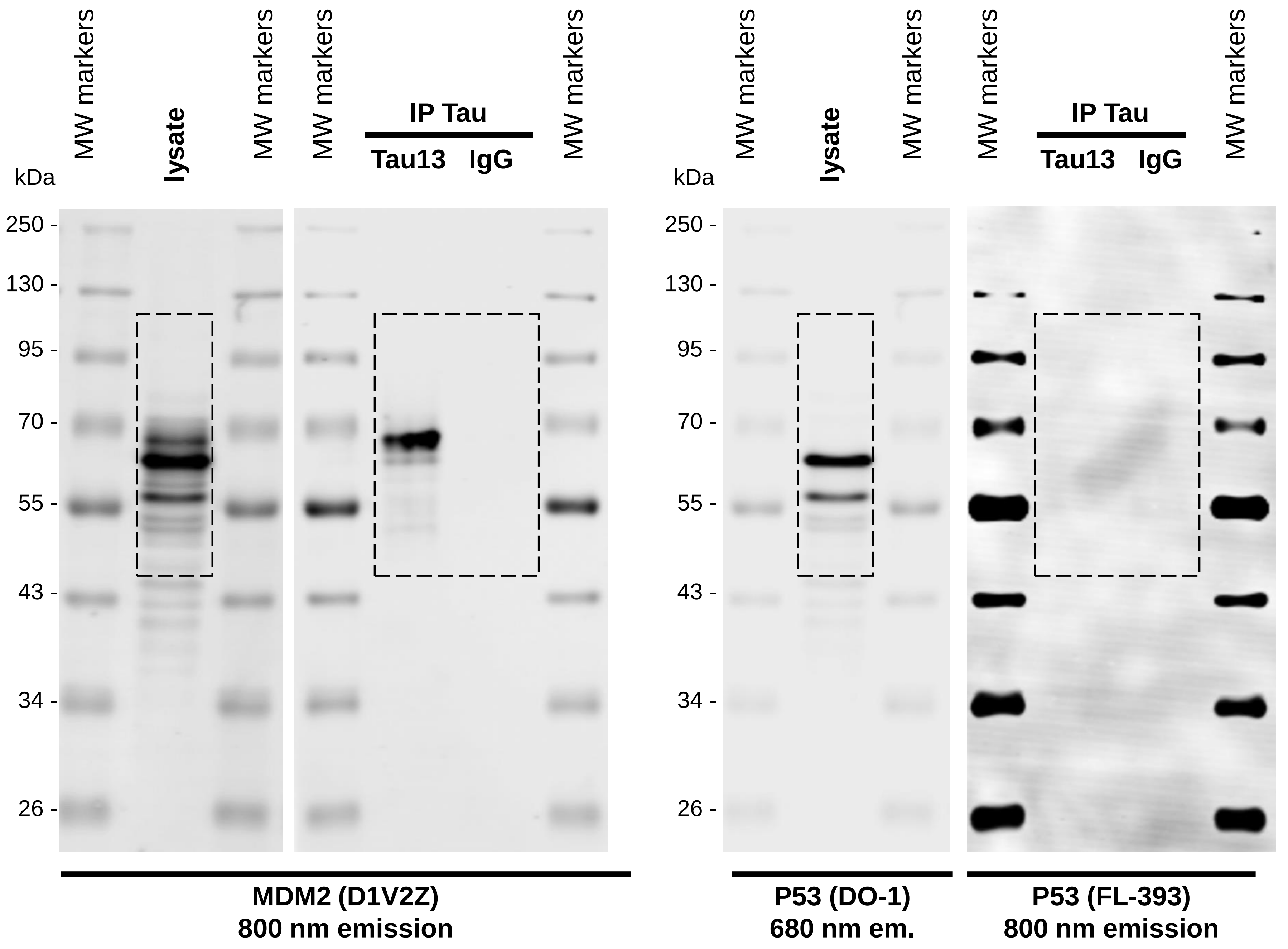

FIGURE 2C raw data MDM2 and Tau WB

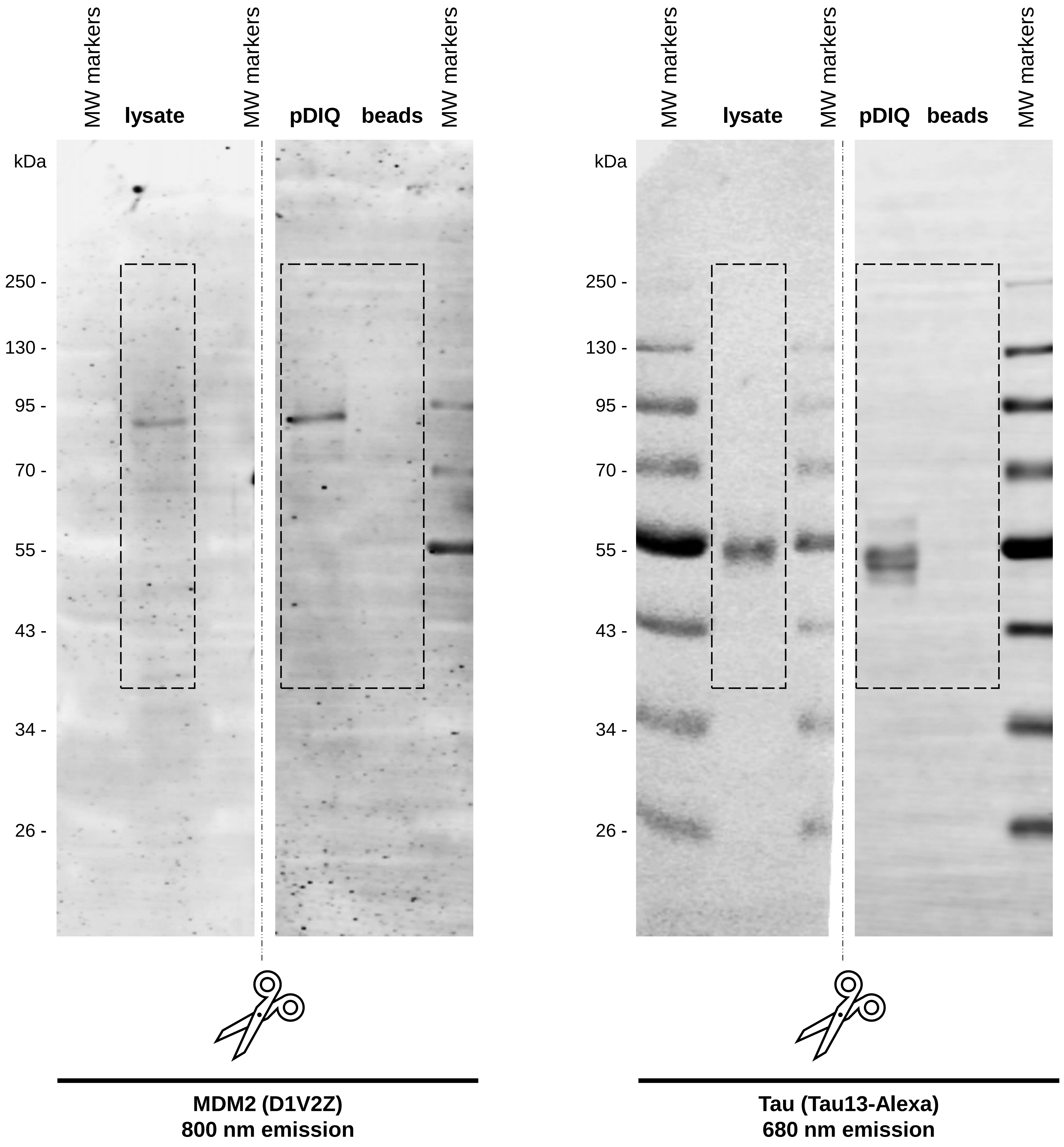

FIGURE 3A raw data MDM2 fragments (cell lysates)

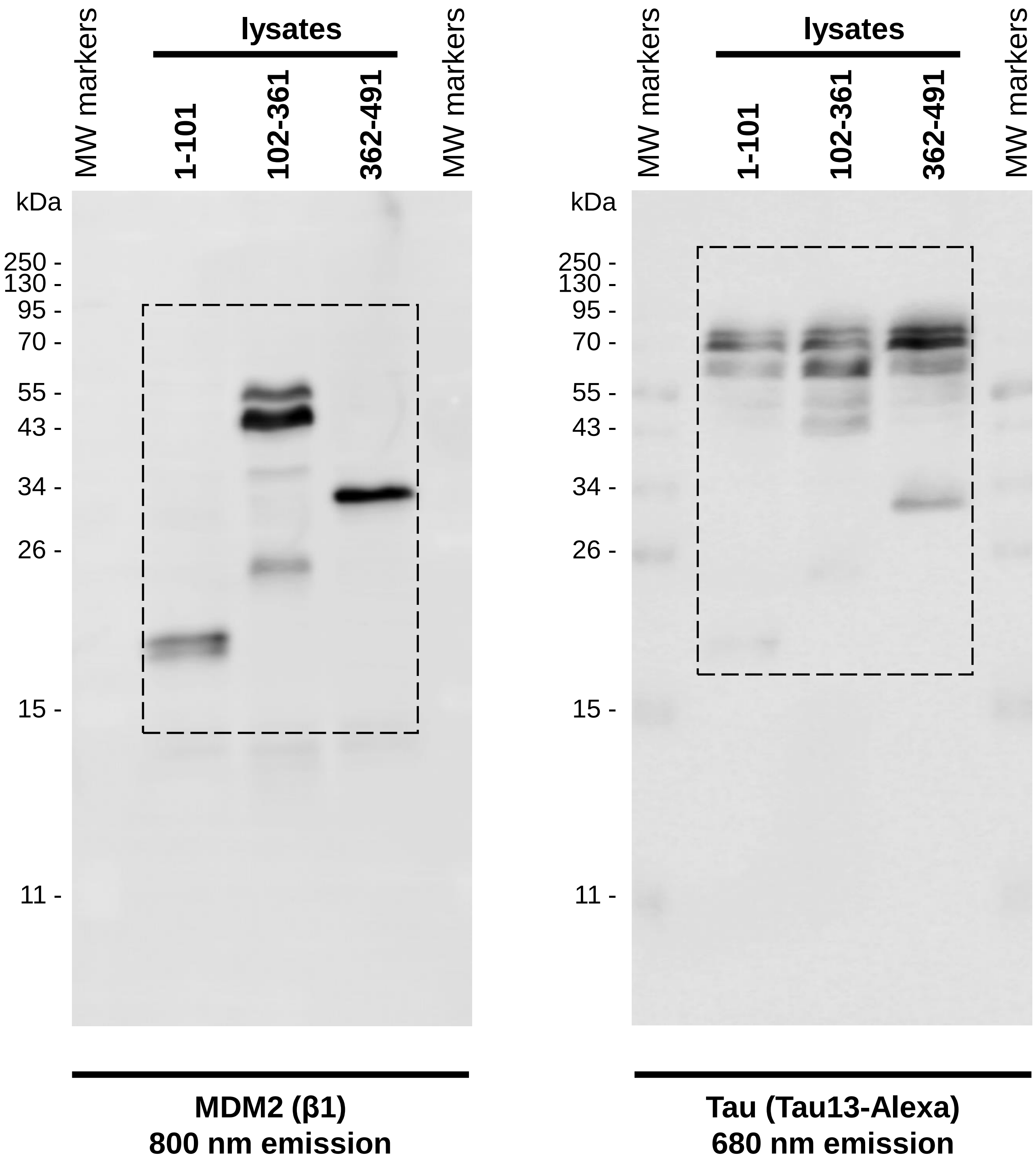

FIGURE 3A raw data MDM2 fragments (IP)

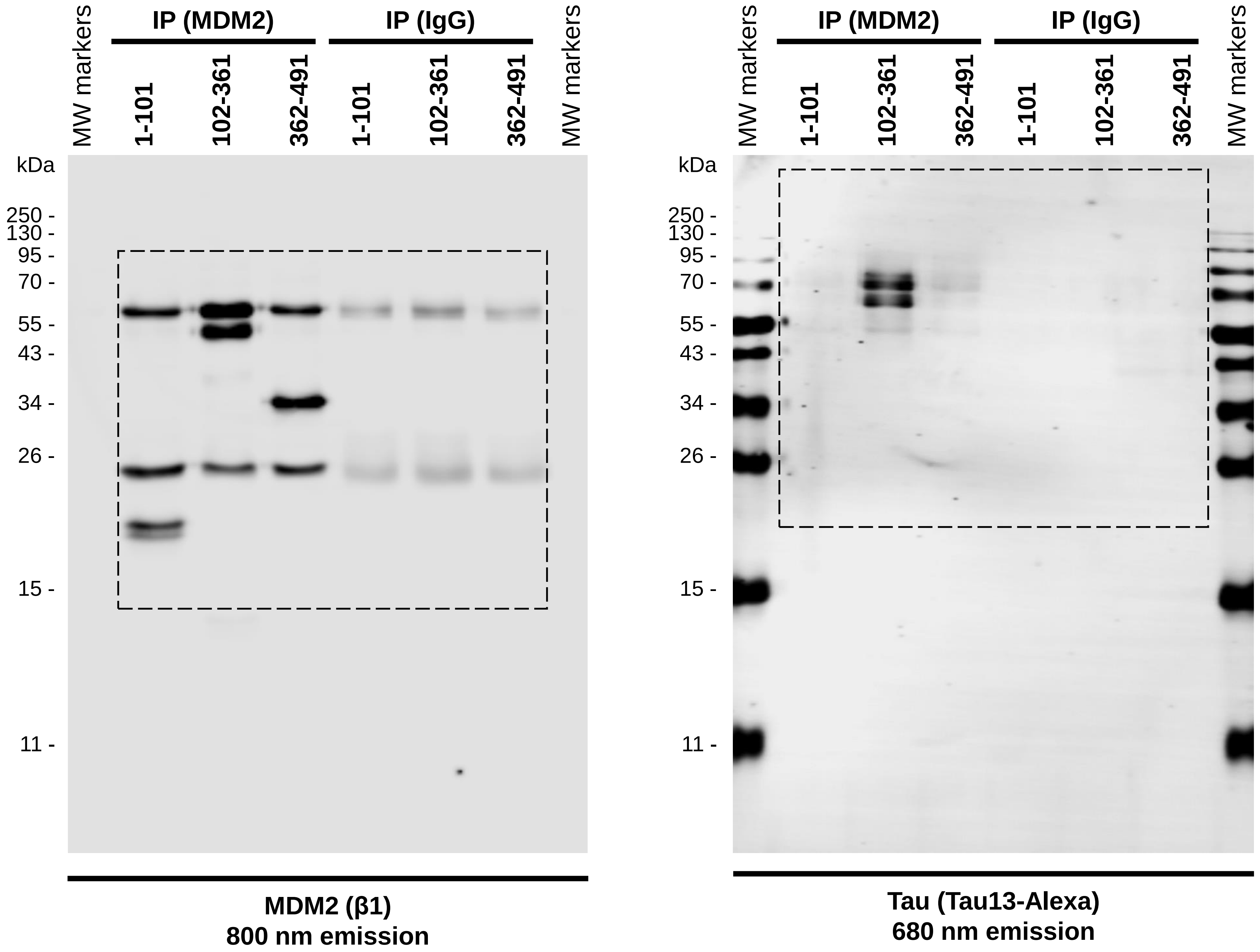

FIGURE 3B raw data Tau fragments (cell lysates)

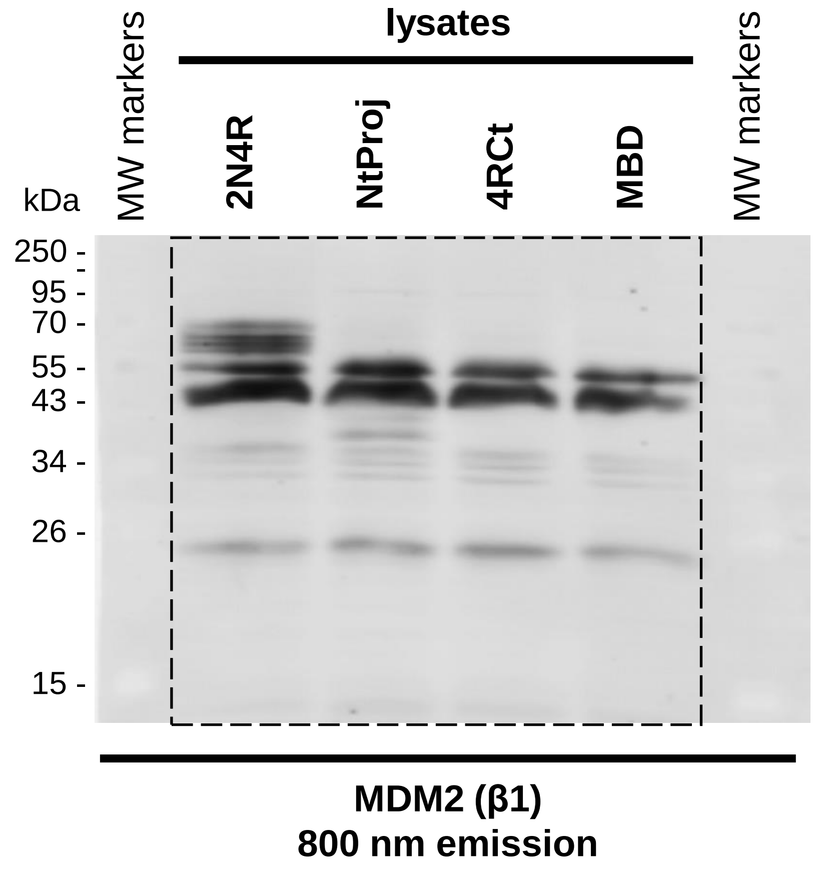

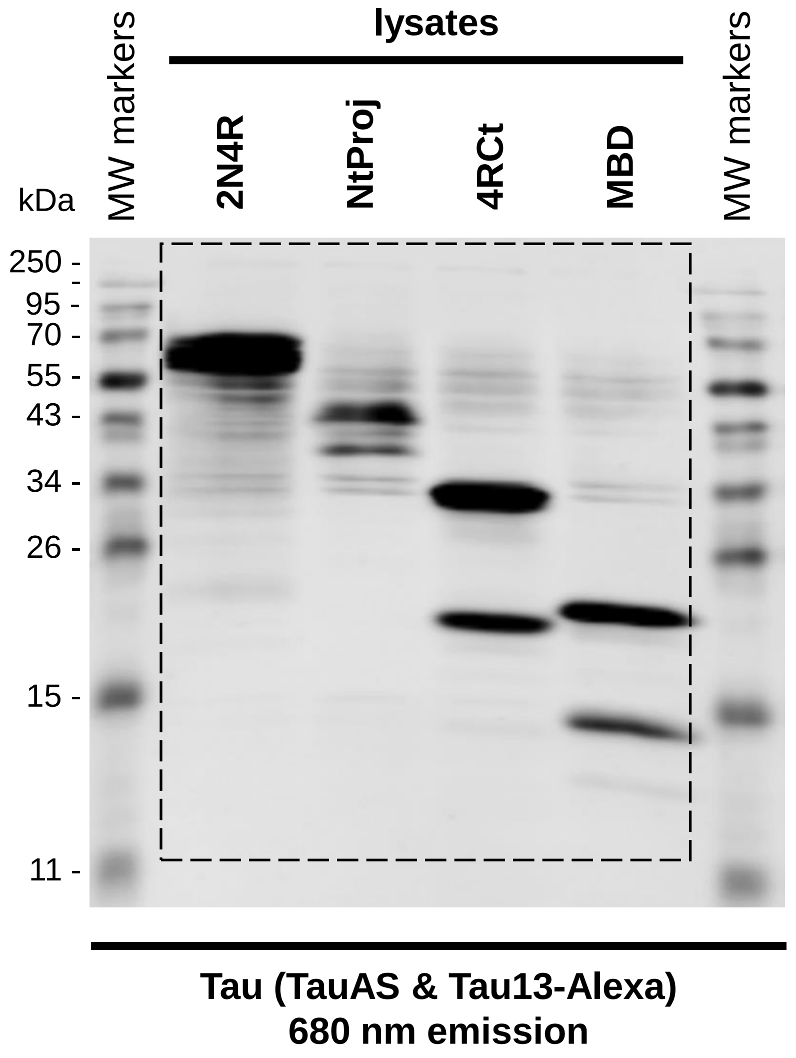

FIGURE 3B raw data Tau fragments (IP)

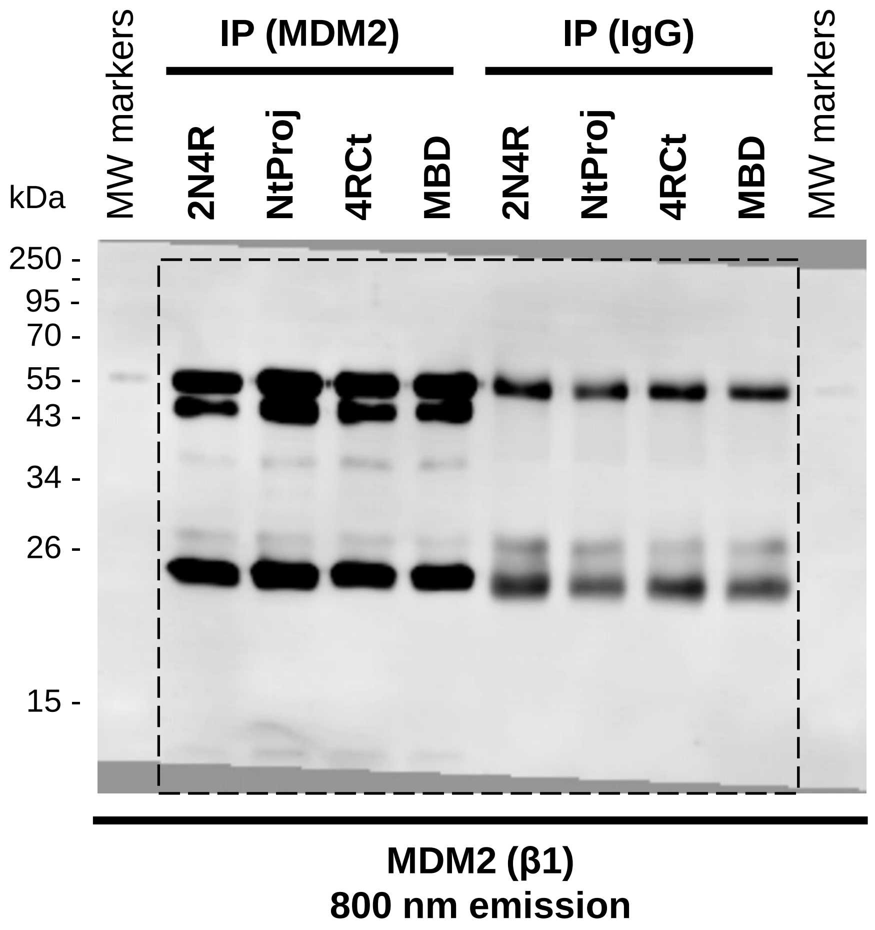

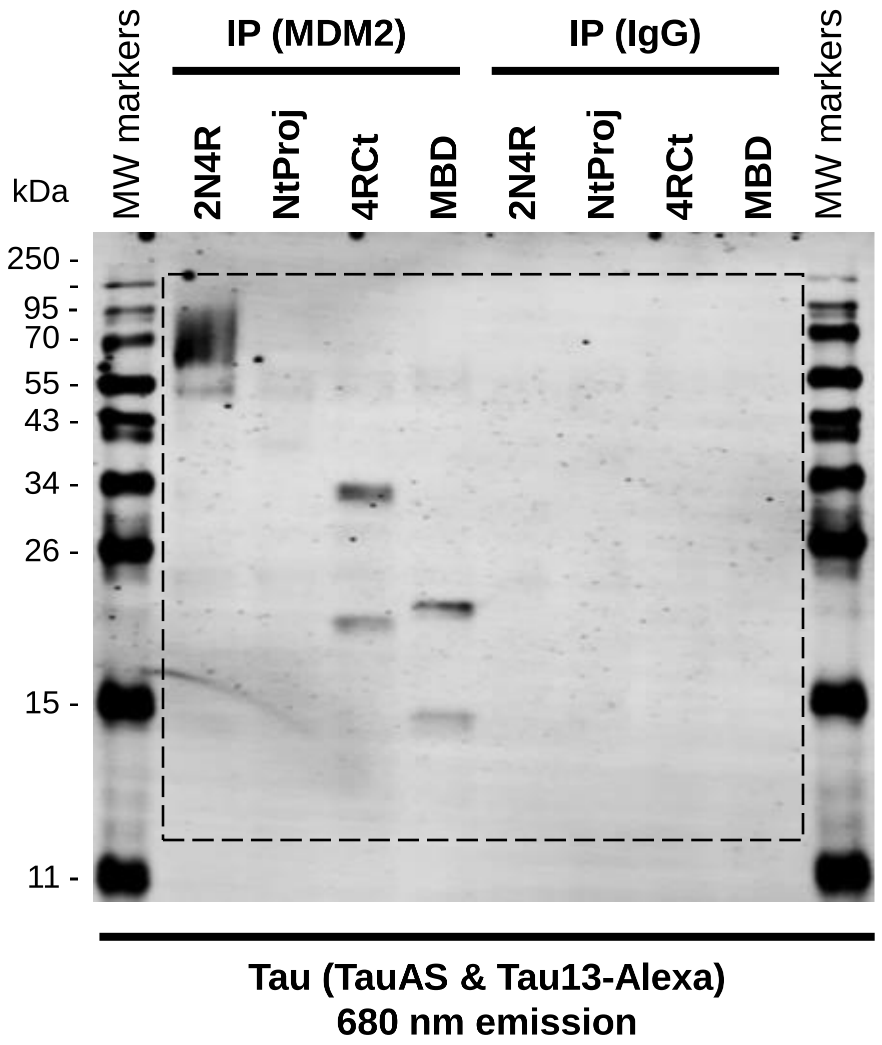

FIGURE 5A raw data MDM2-ubiquitination of P53

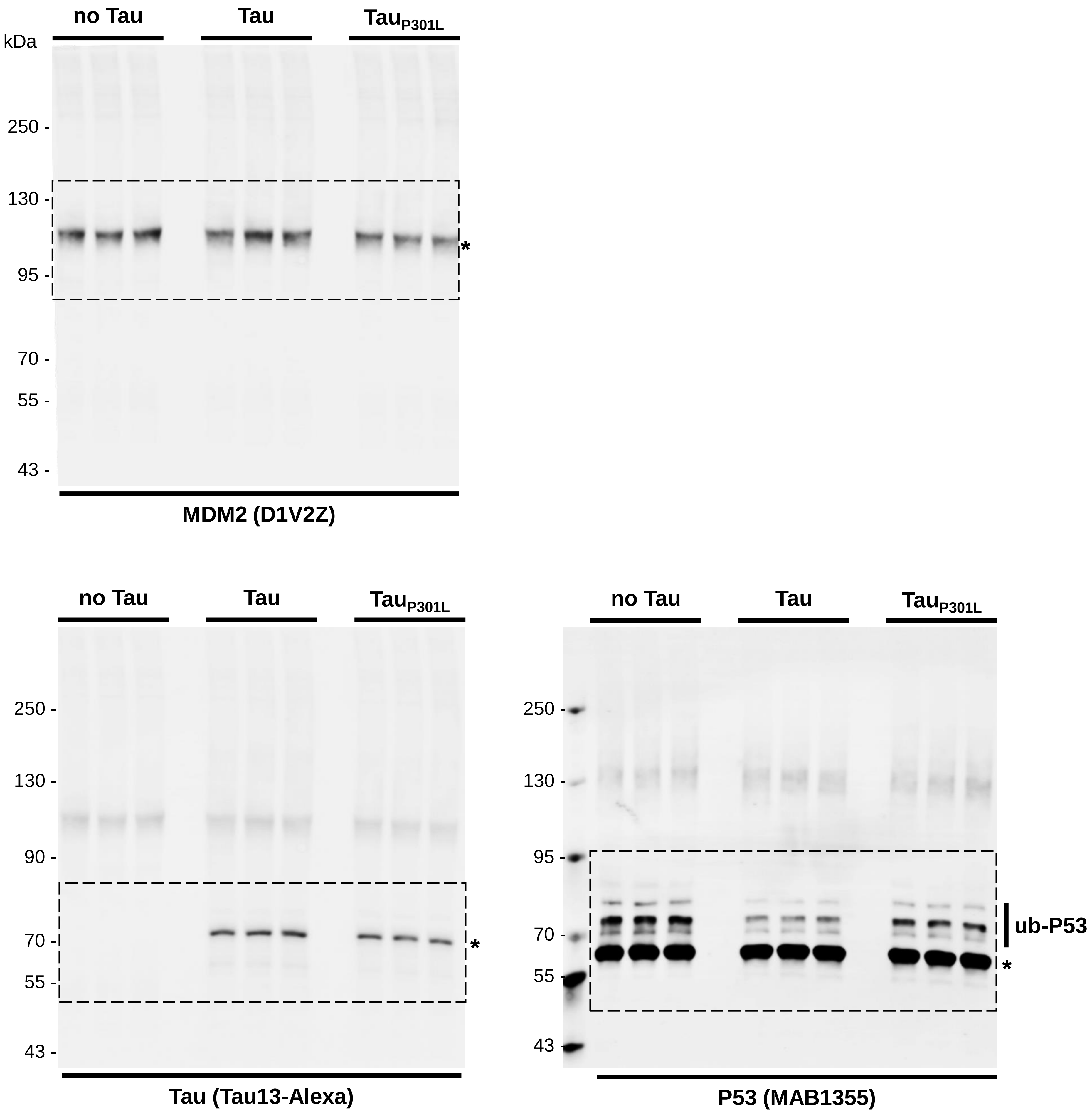

Supplementary Figure 1A raw data

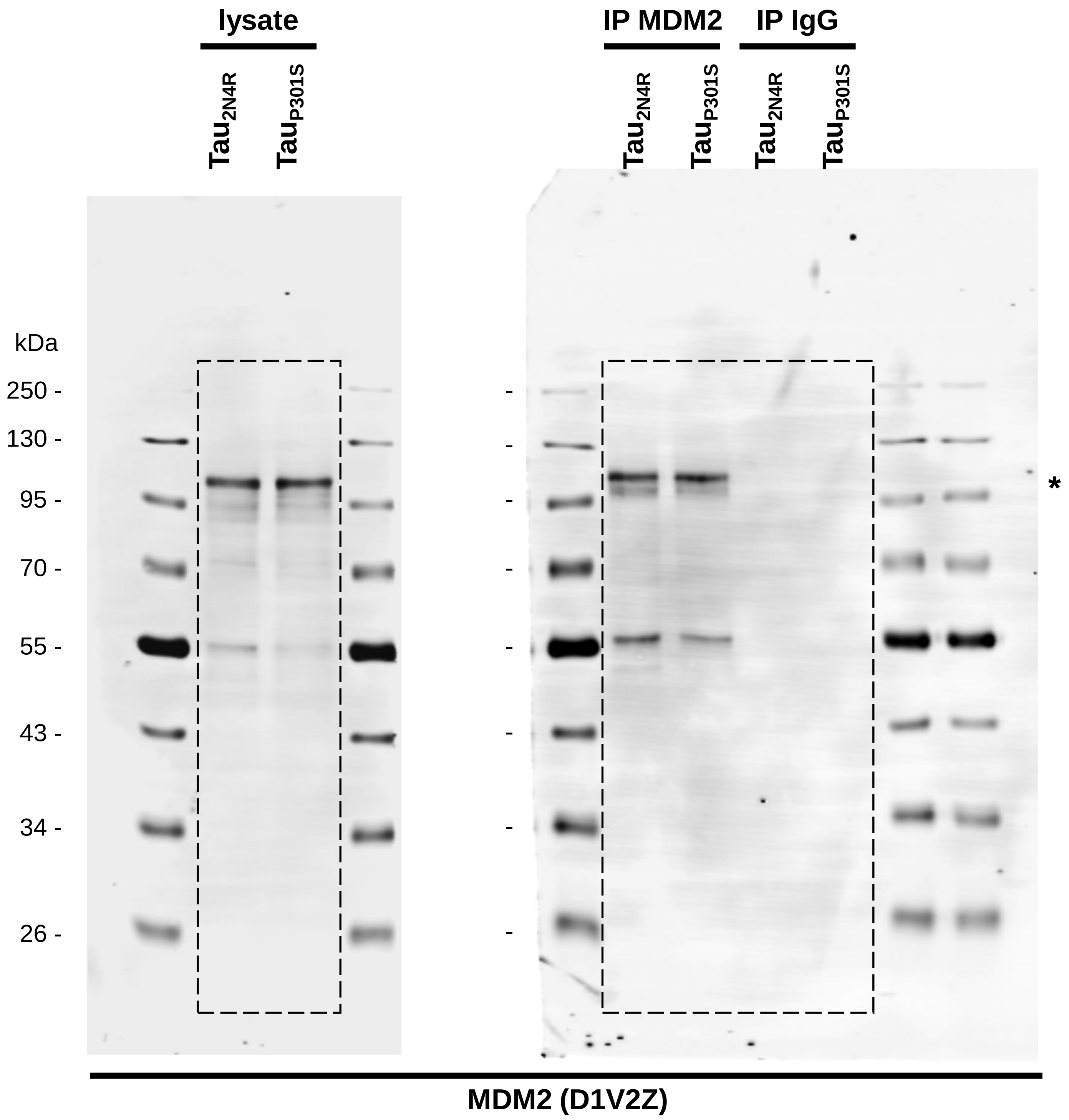

Supplementary Figure 1B raw data

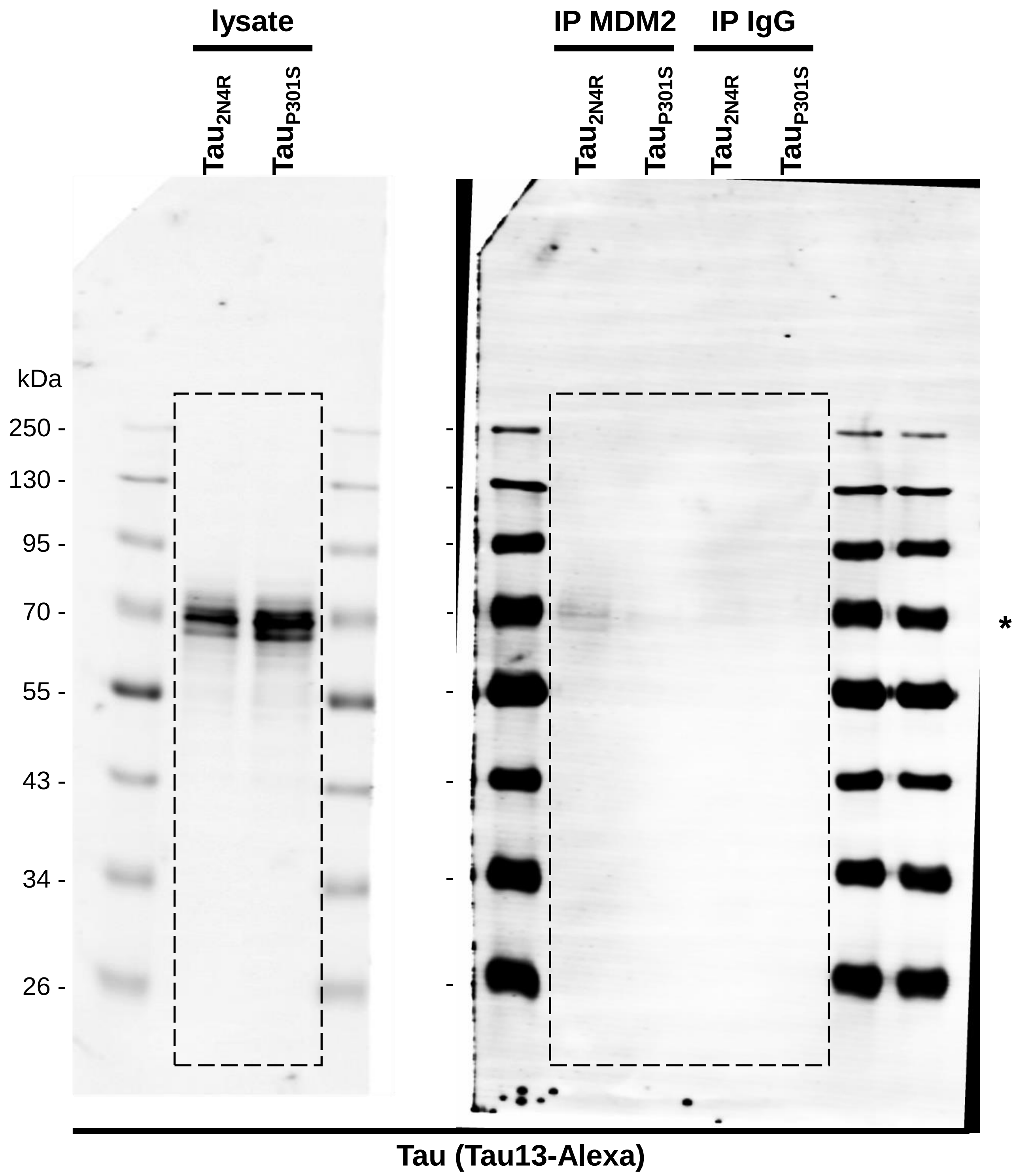

Supplement: Supplementary file 1 — Supplementary Figures. [file 41598_2023_37046_MOESM1_ESM.pdf]
