# Supplementary material for: Tau protein binds to the P53 E3 ubiquitin ligase MDM2
Source: Sci Rep. 2023 Jun 23;13:10208. doi: 10.1038/s41598-023-37046-8 (PMC10290082; doi:10.1038/s41598-023-37046-8)
Supplement: Supplementary file 3 — Supplementary Figures. [file 41598_2023_37046_MOESM3_ESM.docx]

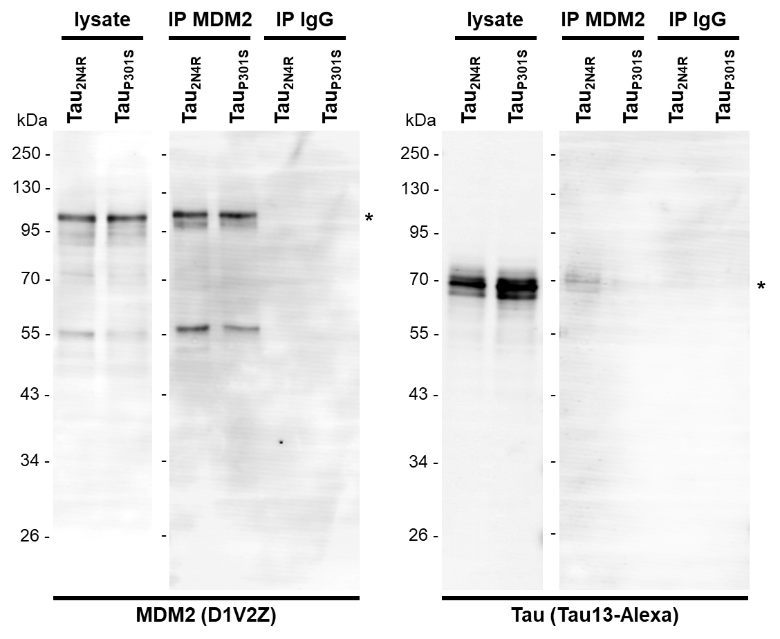


***Supplementary Figure 1. The FTLD mutation P301S impairs the interaction of Tau with MDM2 in cells.*** *Lysates were prepared from Hela cells with ectopic expression of T_11_β1-MDM2 and Tau_2N4R_-S_11_ or Tau_P301S_-S_11_ as indicated. MDM2 was immune precipitated (IP) with matched amounts of the mouse monoclonal β1 antibody or control IgG mouse antibody and samples were resolved on a single gel and analyzed by western blot with either the anti-MDM2 rabbit D1V2Z antibody and anti-rabbit IgG IRDye 800CW, or with the anti-Tau mouse Tau13-AF680 antibody. Molecular weight markers are given on the left of the blots. Original blots are presented in supplementary material (Raw WB Sola).*


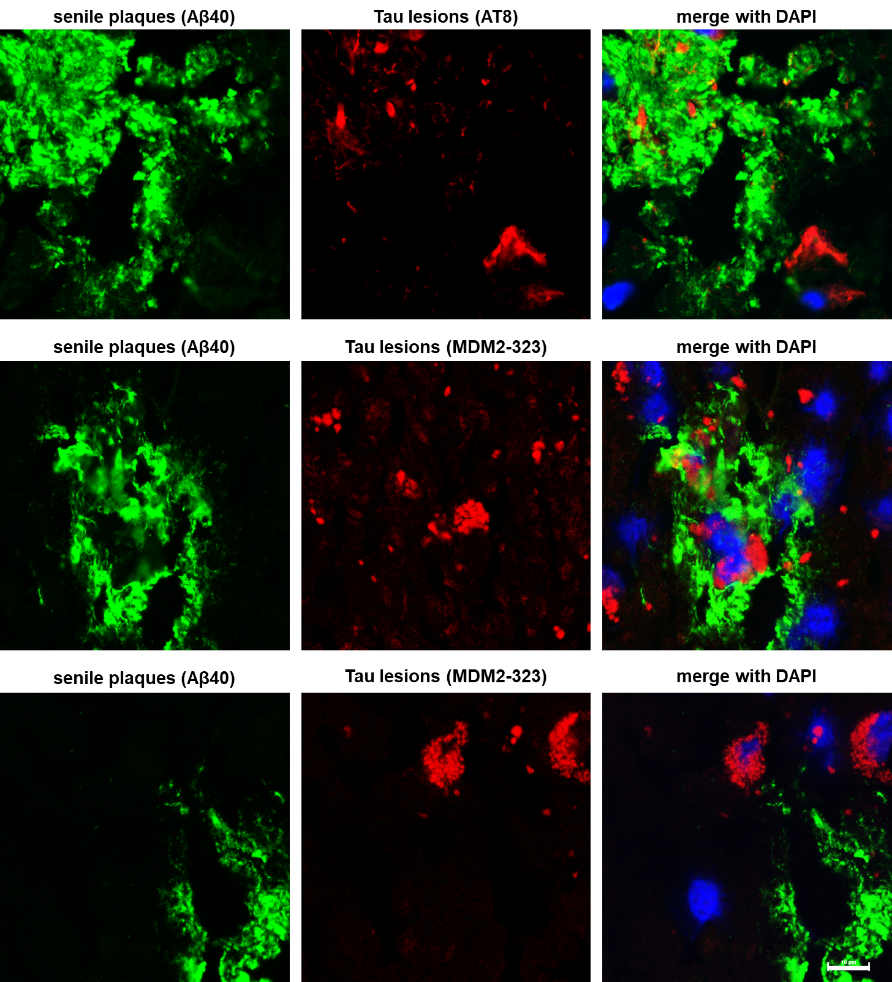


***Supplementary Figure 2. Abnormal MDM2 accumulation in Tau neurofibrillary tangles in the AD brain.*** *Frozen frontal cortex from AD brain sections were stained with the polyclonal rabbit antibody against the C-terminus of Aβ40 and the mouse antibody AT8 against phosphorylated Tau to visualize senile plaques. And Tau lesions (upper row) or the mouse antibody MDM2-323 against MDM2 (lower rows). Merged images include nuclear counterstaining with DAPI. Calibration bar = 10 µm.*
