# Supplementary material for: Tau protein binds to the P53 E3 ubiquitin ligase MDM2
Source: Sci Rep. 2023 Jun 23;13:10208. doi: 10.1038/s41598-023-37046-8 (PMC10290082; doi:10.1038/s41598-023-37046-8)
Supplement: Supplementary file 4 — Supplementary Tables. [file 41598_2023_37046_MOESM4_ESM.pdf]

Supplementary Table 1: cDNA encoding the protein fragments

Protein fragments	cDNA sequence
MDM2 362-491	ATGGAGAAGAGGGACCACATGGTGCTGCTGGAGTACGTGACCGCCGC<br>CGGCATCACCGACGCCTCGGGGGTACCAGGTTCAGAGTTCAGGCACG<br>ACAGCGGCGGACCCGGGAGCGGCGGTGAGGGCTCAGCCGGCGGAGGA<br>CCGGTCGGAGGCGGATCCGCCACCATGTGTAAAAAAACTATAGTGAA<br>TGATTCCAGAGAGTCATGTGTTGAGGAAAATGATGATAAAATTACAC<br>AAGCTTCACAATCACAAGAAAGTGAAGACTATTCTCAGCCATCAACT<br>TCTAGTAGCATTATTTATAGCAGCCAAGAAGATGTGAAAGAGTTTGA<br>AAGGGAAGAAACCCAAGACAAAGAAGAGAGTGTGGAATCTAGTTTGC<br>CCCTTAATGCCATTGAACCTTGTGTGATTTGTCAAGGTCGACCTAAA<br>AATGGTTGCATTGTCCATGGCAAAACAGGACATCTTATGGCCTGCTT<br>TACATGTGCAAAGAAGCTAAAGAAAAGGAATAAGCCCTGCCCAGTAT<br>GTAGACAACCAATTCAAATGATTGTGCTAACTTATTTCCCCTAG
MDM2 102-361	ATGGAGAAGAGGGACCACATGGTGCTGCTGGAGTACGTGACCGCCGC<br>CGGCATCACCGACGCCTCGGGGGTACCAGGTTCAGAGTTCAGGCACG<br>ACAGCGGCGGACCCGGGAGCGGCGGTGAGGGCTCAGCCGGCGGAGGA<br>CCGGTCGGAGGCGGATCCGCCACCATGATCTACAGGAACTTGGTAGT<br>AGTCAATCAGCAGGAATCATCGGACTCAGGTACATCTGTGAGTGAGA<br>ACAGGTGTCACCTTGAAGGTGGGAGTGATCAAAAGGACCTTGTACAA<br>GAGCTTCAGGAAGAGAAACCTTCATCTTCACATTTGGTTTCTAGACC<br>ATCTACCTCATCTAGAAGGAGAGCAATTAGTGAGACAGAAGAAAATT<br>CAGATGAATTATCTGGTGAACGACAAAGAAAACGCCACAAATCTGAT<br>AGTATTTCCCTTTCCTTTGATGAAAGCCTGGCTCTGTGTGTAATAAG<br>GGAGATATGTTGTGAAAGAAGCAGTAGCAGTGAATCTACAGGGACGC<br>CATCGAATCCGGATCTTGATGCTGGTGTAAGTGAACATTCAGGTGAT<br>TGGTTGGATCAGGATTCAGTTTCAGATCAGTTTAGTGTAGAATTTGA<br>AGTTGAATCTCTCGACTCAGAAGATTATAGCCTTAGTGAAGAAGGAC<br>AAGAACTCTCAGATGAAGATGATGAGGTATATCAAGTTACTGTGTAT<br>CAGGCAGGGGAGAGTGATACAGATTCATTTGAAGAAGATCCTGAAAT<br>TTCCTTAGCTGACTATTGGAAATGCACTTCATGCAATGAAATGAATC<br>CCCCCCTTCCATCACATTGCAACAGATGTTGGGCCCTTCGTGAGAAT<br>TGGCTTCCTGAAGATAAAGGGAAAGATAAAGGGGAAATCTCTGAGAA<br>AGCCAAACTGGAAAACTCAACACAAGCTGAAGAGGGCTTTGATGTTC<br>CTGATTAG
MDM2 1-101	ATGGAGAAGAGGGACCACATGGTGCTGCTGGAGTACGTGACCGCCGC<br>CGGCATCACCGACGCCTCGGGGGTACCAGGTTCAGAGTTCAGGCACG<br>ACAGCGGCGGACCCGGGAGCGGCGGTGAGGGCTCAGCCGGCGGAGGA<br>CCGGTCGGAGGC
TAU 1-243	ATGGATCTCCCAGACGATCATTACCTGTCCACCCAGACAATCCTGAG<br>CAAAGATCTTAATGGGGTACCAGGTTACCCATACGATGTTCCAGATT<br>ACGCTGGACCTAGCGGCGGTGAGGGCTCAGCCGGCGGAGGACCGGTC<br>GGAGGCGGATCCACCATGGCTGAGCCCCGCCAGGAGTTCGAAGTGAT<br>GGAAGATCACGCTGGGACGTACGGGTTGGGGGACAGGAAAGATCAGG<br>GGGGCTACACCATGCACCAAGACCAAGAGGGTGACACGGACGCTGGC<br>CTGAAAGAATCTCCCCTGCAGACCCCCACTGAGGACGGATCTGAGGA<br>ACCGGGCTCTGAAACCTCTGATGCTAAGAGCACTCCAACAGCGGAAG<br>ATGTGACAGCACCCTTAGTGGATGAGGGAGCTCCCGGCAAGCAGGCT

	GCCGCGCAGCCCCACACGGAGATCCCAGAAGGAACCACAGCTGAAGA<br>AGCAGGCATTGGAGACACCCCCAGCCTGGAAGACGAAGCTGCTGGTC<br>ACGTGACCCAAGCTCGCATGGTCAGTAAAAGCAAAGACGGGACTGGA<br>AGCGATGACAAAAAAGCCAAGGGGGCTGATGGTAAAACGAAGATCGC<br>CACACCGCGGGGAGCAGCCCCTCCAGGCCAGAAGGGCCAGGCCAACG<br>CCACCAGGATTCCAGCAAAAACCCCGCCCGCTCCAAAGACACCACCC<br>AGCTCTGGTGAACCTCCAAAATCAGGGGATCGCAGCGGCTACAGCAG<br>CCCCGGCTCCCCAGGCACTCCCGGCAGCCGCTCCCGCACCCCGTCCC<br>TTCCAACCCCACCCACCCGGGAGCCCAAGAAGGTGGCAGTGGTCCGT<br>ACTCCACCCAAGTCGCCGTCTTCCGCCAAGAGCCGCCTGTGA
TAU 244-441	ATGGATCTCCCAGACGATCATTACCTGTCCACCCAGACAATCCTGAG<br>CAAAGATCTTAATGGGGTACCAGGTTACCCATACGATGTTCCAGATT<br>ACGCTGGACCTAGCGGCGGTGAGGGCTCAGCCGGCGGAGGACCGGTC<br>GGAGGCGGATCCACCATGCAGACAGCCCCCGTGCCCATGCCAGACCT<br>GAAGAATGTCAAGTCCAAGATCGGCTCCACTGAGAACCTGAAGCACC<br>AGCCGGGAGGCGGGAAGGTGCAGATAATTAATAAGAAGCTGGATCTT<br>AGCAACGTCCAGTCCAAGTGTGGCTCAAAGGATAATATCAAACACGT<br>CCCGGGAGGCGGCAGTGTGCAAATAGTCTACAAACCAGTTGACCTGA<br>GCAAGGTGACCTCCAAGTGTGGCTCATTAGGCAACATCCATCATAAA<br>CCAGGAGGTGGCCAGGTGGAAGTAAAATCTGAGAAGCTTGACTTCAA<br>GGACAGAGTCCAGTCGAAGATTGGGTCCCTGGACAATATCACCCACG<br>TCCCTGGCGGAGGAAATAAAAAGATTGAAACCCACAAGCTGACCTTC<br>CGCGAGAACGCCAAAGCCAAGACAGACCACGGGGCGGAGATCGTGTA<br>CAAGTCGCCAGTGGTGTCTGGGGACACGTCTCCACGGCATCTCAGCA<br>ATGTCTCCTCCACCGGCAGCATCGACATGGTAGACTCGCCCCAGCTC<br>GCCACGCTAGCTGACGAGGTGTCTGCCTCCCTCGCGAAGCAGGGTTT<br>GTGA
TAU 244-372	ATGGATCTCCCAGACGATCATTACCTGTCCACCCAGACAATCCTGAG<br>CAAAGATCTTAATGGGGTACCAGGTTACCCATACGATGTTCCAGATT<br>ACGCTGGACCTAGCGGCGGTGAGGGCTCAGCCGGCGGAGGACCGGTC<br>GGAGGCGGATCCACCATGCAGACAGCCCCCGTGCCCATGCCAGACCT<br>GAAGAATGTCAAGTCCAAGATCGGCTCCACTGAGAACCTGAAGCACC<br>AGCCGGGAGGCGGGAAGGTGCAGATAATTAATAAGAAGCTGGATCTT<br>AGCAACGTCCAGTCCAAGTGTGGCTCAAAGGATAATATCAAACACGT<br>CCCGGGAGGCGGCAGTGTGCAAATAGTCTACAAACCAGTTGACCTGA<br>GCAAGGTGACCTCCAAGTGTGGCTCATTAGGCAACATCCATCATAAA<br>CCAGGAGGTGGCCAGGTGGAAGTAAAATCTGAGAAGCTTGACTTCAA<br>GGACAGAGTCCAGTCGAAGATTGGGTCCCTGGACAATATCACCCACG<br>TCCCTGGCGGAGGAAATAAGATTGAATAA

Supplementary Table 2: PCR Primers (all specific for homo-sapiens mRNAs)

Gene	Forward primer (5'-3')	Reverse primer (5'-3')
MDM2 362-491	GCTCGGATCCGCCACCATGTGTAAAAAAACTATAGTGGAACCAGGATGGC	TGCAGAATTCCTAGGGGAAATAAG
MDM2 102-361	TATAGGATCCGCCACCATGATCTACAGGAACTTGG	GGAGTGAATTCTAATCAGGAACATCAAAGCCC
MDM2 1-101	AGGCGGATCCGCCACCATGTGCAA	GCAGAATTCCTAGGTATATATTTTCCTGTGCTC
MAPT 1-243	GATCAGGATCCACCATGGCTGAG	CATGCTCGAGTCACAGGCGGCTCTTGGCGG
MAPT 244-441	AGGCGGATCCACCATGCAGACAGCCCCCGT	TGATCCTCGAGTCACAAACCCTG
MAPT 244-372	GATCAGGATCCACCATGCAGACA	TGATCCTCGAGTTATTCAATCTT
